# Electrical Equivalent Circuit Parameter Estimation of Commercial Induction Machines Using an Enhanced Grey Wolf Optimization Algorithm

**DOI:** 10.3390/biomimetics10040228

**Published:** 2025-04-06

**Authors:** Premkumar Manoharan, Sowmya Ravichandran, Jagarapu S. V. Siva Kumar, Mustafa Abdullah, Tan Ching Sin, Tengku Juhana Tengku Hashim

**Affiliations:** 1 Institute of Power Engineering (IPE), Department of Electrical and Electronics Engineering, College of Engineering, Universiti Tenaga Nasional (UNITEN), Putrajaya, Kajang 43000, Selangor, Malaysia; 2 Department of Electrical and Electronics Engineering, Dayananda Sagar College of Engineering, Bengaluru 560078, Karnataka, India; 3 Department of Electronics and Communication Engineering, Manipal Institute of Technology Bengaluru, Manipal Academy of Higher Education, Manipal 576104, Karnataka, India; 4 Department of Electrical and Electronics Engineering, GMR Institute of Technology, Rajam 532127, Andhra Pradesh, India; sivakumar.jsv@gmrit.edu.in; 5 Electric Vehicle Engineering Department, Hourani Center for Applied Scientific Research, Faculty of Engineering, Al-Ahliyya Amman University, Amman 19328, Jordan; mrashied@ammanu.edu.jo

**Keywords:** adaptive weight, energy, grey wolf optimizer, induction motor, multimodal optimization, parameter estimation

## Abstract

This paper addresses the critical challenge of optimizing the energy efficiency of induction motors, which are pivotal components across diverse industrial sectors due to their substantial energy consumption. Given the non-measurable internal parameters of induction motors, parameter identification becomes a complex, multidimensional optimization problem characterized by highly nonlinear and multimodal error surfaces. Traditional optimization algorithms often weaken, yielding suboptimal results due to an inadequate balance between the exploration and exploitation phases. To overcome these limitations, this study introduces an Adaptive Weight Grey Wolf Optimizer (AWGWO) to enhance the accuracy and reliability of induction motor parameter estimation. The AWGWO incorporates an adaptive weight mechanism that dynamically adjusts the exploration and exploitation balance, effectively mitigating issues such as premature convergence to local optima. Extensive simulation validation was conducted across various induction motor models, including eight commercial motors, and demonstrated that AWGWO consistently outperforms state-of-the-art algorithms in terms of convergence speed, solution accuracy, and robustness in multimodal optimization landscapes. The AWGWO consistently exhibited faster convergence, significantly reducing premature convergence. Moreover, the adaptive weight mechanism enabled a more effective balance between exploration and exploitation, leading to higher accuracy in parameter estimation. Comparative analyses reveal that AWGWO outperforms existing algorithms not only in achieving lower error rates, but also in maintaining stability. This study significantly contributes to progress in the field by providing an effective tool for induction motor parameterization, thereby offering potential improvements in energy efficiency.

## 1. Introduction

### 1.1. Basic Concepts

The power components of the electrical power system, such as transformers, breakers, relays, etc., must operate reliably to deliver continuous power to consumers. On the other hand, induction motors (IMs) must operate consistently to enable smooth manufacturing processes. The functionality of the essential components, on the other hand, deteriorates as a result of various external conditions. As a result, continual evaluation of such components is essential in order to guarantee that they are performing satisfactorily [1]. Electrical machines’ behaviour and performance are characterized by the equivalent circuit characteristics used in their design. Since the machine’s condition and performance are dependent on the assessment of equivalent circuit parameters, this can provide crucial data on its state and effectiveness [2]. Machine modelling is extremely crucial for state-of-the-art current AC drive systems, such as speed control of induction motors, which are a good example of this. If users want to use a control algorithm to operate induction motors, users must first determine the rotor flux linkage assessment, requiring a reliable motor analogous electrical parameters assessment. In accordance with IEEE standards, the equivalent circuit characteristics of induction machines can be evaluated using a conventional offline testing methods. However, these methods cannot be applied to machines that are already in operation.. Aside from that, deviations in ambient circumstances (such as temperature, humidity, and so on) might result in a change in the equivalent circuit characteristics. Because of this, the current research focuses on measuring the state of those components while they are connected to the power network [3].

The squirrel cage induction machines are perhaps the most popular rotating machine used in most of the industries today. Compared to other rotating machines, these machines offer numerous benefits, such as simple operation, simple maintenance, affordable pricing, small size, high performance, etc. Therefore, IMs are widely regarded as the “powerhouse motors” of their respective industries [4]. Among the many various uses for such machines are those that operate at variable speeds, with variable loads, and operate at constant or variable voltage supplies. Although its parameters should be calculated with high accuracy, they must be estimated with high precision in order to investigate and model the IM’s behaviour (including voltage drop estimates, load variation calculations, system analysis, frequency response, and so on). Consequently, a reliable and accurate parameter estimate approach and a sufficient equivalent circuit are necessary for this context. As a result, this issue has been addressed in the major international standards and research papers that explore the standards mentioned above [5]. Numerous research studies have been conducted to estimate analogous electrical characteristics for IMs, and the results have been published. An interactive dynamic parameter estimation method based on the recursive least square (RLS) algorithm is described in the paper [6]. Another induction motor parameter estimation strategy, devised by [2], is a recursive least square algorithm-based approach. An online parameter identification system for induction motors was also presented, which was based on the RLS algorithm, which was based on [7]. It was discovered that they had solved the Butterworth digital filtering model using an improved Euler’s numerical solution, allowing them to enhance the prediction quality. The authors of [8] were the first to propose applying soft computing methods to diagnose electrical equipment. The study in [9] presented an automatic procedure for estimating induction motor parameters at a standstill. It presents a novel algorithm that utilizes standstill data for parameter estimation, bypassing the need for dynamic operation. The study highlights the method’s efficiency and accuracy, providing a practical solution for motor parameter identification without requiring operational testing.

### 1.2. Related Works

Various IM parameter estimation methods are available in the literature, which can be classified into various methods [10,11]. In addition, the authors also provide a survey of estimate methods, with a particular emphasis on their applicability to machine learning problems. Using the classification scheme proposed by [12], methods for the characterization of IM model parameters can be divided into five classifications: methods based on steady-state machine models, machine structure data, real-time parameter identification, time-domain parameter identification, and frequency-domain parameter identification. The authors of [13] reported a two-stage optimization approach to estimate the parameters of the induction motor. The formulation of an optimization problem aimed to minimize the objective function by optimizing the decision variables and the data provided by the manufacturer. The authors of [11] developed a method for estimating the parameters of a three-phase IM solely from nameplate data using the Gauss–Seidel algorithm. However, such approaches are hampered by the inability to pick appropriate boundary conditions and variables with changes in ambient conditions, ageing, and rewinding. The authors proposed a technique, as stated in [14], to derive the circuit variables for induction motors with the National Electrical Manufacturers Association design. The approach employed by the researchers was founded upon developing a collection of nonlinear equations obtained from the equivalent circuit of the induction machine, utilizing data provided by the manufacturers. In [15], a seven-parameter equivalent circuit was introduced to depict the rotor through the utilization of two resistive-inductive branches that are connected in parallel. According to the report published in the paper, the parameter estimation indicates that the resistance of the stator holds the least significance.

The study in [16] investigated various analytical methods for deriving three-phase induction motor equivalent circuit parameters using only nameplate data. It compares the efficacy of different techniques in parameter estimation, demonstrating how each method influences limited information to achieve accurate results. The study highlights the trade-offs and accuracy of these analytical approaches. The authors of [17] introduced a novel method for estimating induction machine parameters using no-load startup transient data. It demonstrates how this approach leverages transient responses to derive accurate parameters, offering an alternative to conventional steady-state methods. The study highlights its effectiveness in capturing dynamic behaviour and improving parameter estimation precision. Genetic algorithm-based IM model parameters were proposed in [18], utilizing a genetic algorithm (GA). According to [19], the authors provided a strategy for IM parameter identification that uses the chaotic ant swarm algorithm and showed that the inaccuracies are minimized compared to the GA. The authors of [20] have suggested a technique for estimating the parameters of a split-phase IM using the Levenberg–Marquardt algorithm. The particle swarm optimizer (PSO)-based evolution optimization method for IM parameter identification was proposed [21]. The study in [22] introduces a hybrid simulated annealing–evaporation rate water cycle algorithm for parameter estimation of single-cage and double-cage induction machine models. By combining these optimization techniques, the algorithm enhances search efficiency and accuracy, effectively minimizing errors in parameter estimation. The study demonstrates its superiority over conventional methods, such as the differential evolution (DE) algorithm, GA, PSO, shuffled frog-leaping algorithm (SFLA), and modified shuffled frog-leaping algorithm (MSFLA), particularly in handling the complex dynamics of induction machine models. The researchers combined a constriction factor with a PSO to determine the characteristics of an IM. To identify the IM variables, the authors developed a modified PSO technique [23]. In [1], a PSO was used to develop a methodology for parameter identification for single-phase transformers and three-phase IMs. The equivalent circuit parameter estimation problem was addressed in [24] by proposing three distinct metaheuristic methods, which were subsequently evaluated on various IMs. The study on the determination of induction motor parameters using the differential evolutionary algorithm reviews various optimization techniques for motor parameterization, emphasizing DE’s robustness in handling complex, nonlinear error surfaces [25,26]. The study highlights DE’s superiority in achieving accurate parameter estimates with enhanced convergence rates and minimal computational overhead compared to traditional methods. The study in [27] explores the integration of Simulated Annealing with Particle Swarm Optimization (SA-PSO) for induction motor parameter identification, leveraging correlation-based techniques to enhance search efficiency. The hybrid algorithm mitigates the risks of premature convergence and local optima entrapment. The algorithm demonstrated improved accuracy and convergence speed in parameter estimation compared to conventional PSO approaches. The authors of [28] presented a GA-based approach for off-line parameter estimation of induction motors, accounting for magnetic saturation and iron losses. The GA effectively optimizes the nonlinear motor model, providing accurate parameter estimation. The study highlights GA’s capability in handling complex, multidimensional optimization challenges, leading to improved model fidelity over traditional methods.

The authors of [29] introduce the Gravitational Search Algorithm (GSA) to determine the parameters of IMs in both approximate and exact circuit models. This is achieved through the use of two distinct induction motors. Estimating parameters for an induction motor is commonly approached as an optimization problem utilizing the least squares methodology. However, due to the nonlinear nature of the steady-state equivalent circuit model, this can present a complex challenge. The authors of [30] introduce a novel approach for parameter estimation of IMs that obviates the requirement of no-load and locked rotor tests. The proposed methodology relies on an optimization framework utilizing the Artificial Bee Colony (ABC) algorithm, which is a recently developed swarm-based optimization technique. Two distinct equivalent circuits have been employed in the parameter estimation methodology. The author of [31] presents a modified version of the ABC algorithm, called Disruption Black Hole ABC (DBHABC), which is inspired by swarm intelligence and incorporates principles from physics for the parameter estimation of IM. The comparison is made with ABC, Black Hole ABC (BHABC), Enhanced ABC, and Best-So-Far ABC (BSFABC). The study in [32] presents a numerical and experimental approach for optimal parameter identification of a three-phase induction motor using advanced optimization techniques. It emphasizes the accuracy of modern algorithms in estimating motor parameters. The algorithms are validated through experimental verification and demonstrate their effectiveness in achieving precise, reliable parameter estimates compared to traditional methods. The study in [33] introduces the Vortex search optimization algorithm for the three-phase IM parameter estimation. The study in [34] proposed a salp swarm optimization algorithm and estimated the parameters of the IM based on the least squares method. The study in [35] introduces a hybrid PSO–Jaya optimization algorithm specifically designed for parameter estimation in poly-phase induction motors, combining the strengths of Jaya, DE, GA, and PSO to enhance accuracy and convergence. The proposed method is experimentally validated, showing superior performance in estimating motor parameters and effectively addressing limitations of single method approaches in complex optimization landscapes. The study in [36] proposes a two-stage PSO algorithm for the accurate parameter estimation of eight commercial induction motors from ABB. By integrating a combined method, the approach effectively refines the search process, enhancing both global and local optimization. Experimental results confirm the method’s ability to deliver precise parameter estimates, outperforming traditional single-stage techniques.

### 1.3. Advanced Techniques for IM Parameter Estimation

The use of machine learning algorithms for the parameter estimation of the induction motor is not well established. Few researchers have applied basic machine learning methods to identify IM parameters. Deep learning and machine learning algorithms are mostly used for speed estimation [37], rotor flux estimation [37], torque estimation [37], resistance estimation [37,38], rotor time constant estimation [37,39], time-varying parameter identification [40], excitation inductance estimation [38], predictive maintenance in induction motors [41], impedance estimation and unbalance supply voltage detection [42], etc. The authors of [43] explored parameter estimation of squirrel-cage induction motors using Artificial Neural Networks (ANN) and Adaptive Neuro-Fuzzy Inference Systems (ANFIS). They compared the effectiveness of these approaches in modelling complex motor behaviours and improving parameter accuracy. The study shows that ANFIS enhances estimation precision beyond traditional ANN methods, offering robust solutions. The authors of [44] explored online parameter estimation of three-phase induction motors using ANNs. The study demonstrated ANNs’ ability to adaptively estimate equivalent circuit parameters in real-time, improving accuracy and efficiency. The study shows that ANNs outperform traditional methods, providing reliable and dynamic parameter estimation for motor diagnostics and control. The study in [45,46] presented an optimal parameter estimation method for induction machines using ANNs. It highlights how ANNs can model complex relationships and enhance the accuracy of parameter estimates. The study emphasizes the advantages of ANNs over traditional methods, particularly in capturing nonlinearities and improving estimation precision. The authors of [47] investigated the parametric estimation of induction motors using torque data optimized through a generalized normal distribution optimizer. It demonstrates how this method improves parameter accuracy by effectively handling torque data’s statistical variations. The study highlights the advantages of enhancing the precision of motor parameter estimation compared to traditional techniques. The study in [48] explored the synergy between electrostatic discharge optimizer and experimental verification for parameter estimation of three-phase induction motors. It highlights how integrating this novel optimization technique with empirical data enhances accuracy and reliability in parameter estimation. The study demonstrates significant improvements over conventional methods in modelling motor performance.

### 1.4. Motivation

The Grey Wolf Optimizer (GWO) [49,50,51,52] is a new metaheuristic algorithm inspired by the hunting behaviour and social hierarchy of the grey wolf. GWO outperforms most existing metaheuristic algorithms in multimodal situations, avoiding drawbacks such as early convergence to sub-optimal solutions, which are common in other optimization algorithms. Because of these qualities have been used to tackle a wide range of engineering problems, including power and energy, image processing, and machine learning, among others. However, due to the drawbacks, such as slow convergence rate, parameter sensitivity, lack of diversity, scalability issues, and adaptability, the researchers have proposed different versions of the GWO algorithm. The detailed study on recent variants of GWO is as follows. The authors of [53] proposed the Adaptive Grey Wolf Optimizer (AGWO), which introduces an adaptive strategy for updating the position vector. This modification aims to improve the exploration and exploitation capabilities of the GWO algorithm. The authors showed that AGWO outperforms the original GWO and other optimization algorithms in solving benchmark functions. The Chaotic Grey Wolf Optimizer (CGWO) was introduced by [54], incorporating chaotic sequences into the original GWO algorithm. These sequences enhance the global search ability and convergence speed. The authors demonstrated the effectiveness of CGWO on various benchmark functions and real-world optimization problems. The Quantum Grey Wolf Optimizer (QGWO) is a hybrid optimization algorithm proposed in [55]. This method combines the advantages of quantum computing and GWO. The quantum behaviour allows the algorithm to explore the search space more effectively and enhances its convergence speed. QGWO has been successfully applied to various optimization problems. The authors of [56] reported a k-means clustering-based GWO algorithm. The algorithm was validated using the benchmark functions and data clustering problems. An Opposition-Based Grey Wolf Optimizer (OGWO) was proposed in [57]. This variant incorporates opposition-based learning, which enhances the algorithm’s global search capability and accelerates convergence. The authors demonstrated the superior performance of OGWO in comparison to the original GWO and other optimization algorithms. The study in [58] reported a multi-learning strategy to improve the performance of the GWO and applied it to 5G uplink communication. The authors of [59] proposed a Grey Wolf Optimizer with local search (LSGWO) that integrates the global search of GWO with a local search strategy. This combination balances exploration and exploitation, leading to faster convergence and improved performance. LSGWO has been successfully applied to various optimization problems. The Improved Grey Wolf Optimizer with Dynamic Search (IGWO-DS) was proposed in [60]. This method incorporates dynamic search strategies based on population-based inertia weight and opposition-based learning. This combination enhances the exploration and exploitation capabilities of the algorithm, resulting in improved performance and faster convergence. IGWO-DS has been successfully applied to various optimization problems. The authors of [61] introduced the Grey Wolf Optimizer with Levy flight (LGWO), which integrates Levy flight into the GWO algorithm. This modification enhances the exploration capability of the algorithm, allowing it to escape local optima and converge faster. The authors demonstrated the superior performance of LGWO compared to the original GWO and other optimization algorithms. The Grey Wolf Optimizer with Nelder–Mead method (NM-GWO) was proposed in [62]. This hybrid optimization algorithm combines the global search capability of GWO with the local search of the Nelder–Mead method. The authors showed that NM-GWO outperforms the original GWO and other optimization algorithms in solving benchmark functions and real-world optimization problems. The authors of [63] introduced a variant of the Grey Wolf Optimizer with crossover and mutation (GWO-CM), which incorporates genetic algorithm operators into the GWO framework. This modification improves the balance between exploration and exploitation, resulting in better performance. GWO-CM has been successfully applied to various optimization problems. A self-adaptive Grey Wolf Optimizer (SAGWO) was proposed in [64]. This variant dynamically adjusts the algorithm’s control parameters, enhancing its search capability and convergence speed. The authors demonstrated the effectiveness of SAGWO in comparison to the original GWO and other optimization algorithms.

The detailed literature study shows that each variant of GWO has merits and drawbacks and has been proposed for different numerical optimization problems and real-world engineering design problems. Nevertheless, none of the GWO variants are applied to parameter estimation of the IM models, including the original version of GWO. To overcome the limitations of the existing GWO algorithm, this study introduces an Adaptive Weight Grey Wolf Optimizer (AWGWO) to enhance the accuracy and reliability of induction motor parameter estimation. The AWGWO incorporates an adaptive weight mechanism that dynamically adjusts the exploration and exploitation balance, effectively mitigating issues such as premature convergence to local optima. The key contributions of this research are as follows:

- The study introduces the GWO algorithm with an adaptive weight mechanism to dynamically balance exploration and exploitation and address the limitations of existing algorithms in parameter estimation of the IM.
- The AWGWO enhances the accuracy of IM parameter estimation by handling multimodal and nonlinear optimization search space, reducing premature convergence.
- The proposed AMGWO is validated using the numerical benchmark problems, which include different features like unimodal and multimodal with variable dimensions.
- The proposed AWGWO is also validated using the IM benchmark model and eight commercial motors to demonstrate the performance compared to other algorithms.

The paper is organized as follows. Section 2 deals with the mathematical modelling and problem formulation of the parameter estimation of the IMs optimization problem. Section 3 discusses the original version of the GWO and also presents the concepts of the proposed AWGWO algorithm. Section 4 comprehensively explains the results and further discussions. Finally, Section 5 concludes the paper.

## 2. Problem Formulation

The characteristics of an IM are not readily observable or quantifiable in nature. As a result, prediction procedures are frequently used to make estimates about entities. In such methods, the behaviour of an IM is mimicked by nonlinear circuits that are equivalent to the IM. The approximate circuit model and the exact circuit model are the two types of circuit models [10] that are available based on the accuracy required. In particular, they enable the proper relationship of the machine parameters to be established to estimate them. It is turned into a multidimensional optimization process during the identification step, in which the intrinsic characteristics of the IM are treated as decision variables. As a result, there are six different parameters in the exact model and four parameters in the approximate model. Nevertheless, in many articles dealing with calculating IM parameter values, the significance of the core loss impedance is overlooked. As a result, the objective is to reduce the difference between the predicted and the manufacturer’s data to the smallest possible amount by modifying the characteristics of the analogous circuit. Under this technique, the complexity of the problem formulations tend to yield multimodal error landscapes for which it is extremely difficult to minimize their fitness functions.

### 2.1. Approximate Model of the Induction Machine

**Figure 1:**
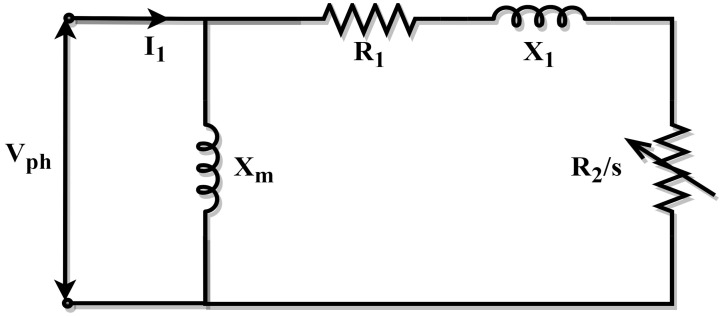
Approximate model of the IM.

Consequently, the approximate circuit model is less accurate than the exact circuit model since it does not consider the rotor reactance and the magnetizing reactance throughout its design. In order to compute the stator resistance ($R_{1}$), stator leakage reactance ($X_{1}$), slip (s), and rotor resistance ($R_{2}$), the approximate circuit model is used in conjunction with the manufacturer’s starting torque ($T_{st}$), maximum torque ($T_{m}$), and full load torque ($T_{fl}$) data. The approximate circuit model is depicted in Figure 1 [65].

The estimation problem can be stated as the following optimization problem under the assumptions of the approximate circuit model:

(1) $$\mathrm{Minimize}\;f\left(x\right),\;x=\left\{R_{1},R_{2},X_{1},s\right\}\in R^{4}$$

Subject to:

(2) $$\left.\begin{array}{c} 0\leq R_{1}\leq 1\\ 0\leq R_{2}\leq 1\\ 0\leq X_{1}\leq 10\\ 0\leq s\leq 1 \end{array}\right\}$$

where

(3) $$f\left(x\right)={f_{1}(x)}^{2}+{f_{2}(x)}^{2}+{f_{3}\left(x\right)}^{2}$$

(4) f1x=KtR2sR1+R2s2+X12−TflTfl

(5) $$f_{2}\left(x\right)=\frac{\frac{K_{t}R_{2}}{{\left(R_{1}+R_{2}\right)}^{2}+X_{1}^{2}}-T_{st}}{T_{st}}$$

(6) $$f_{3}\left(x\right)=\frac{\frac{K_{t}}{2\left[R_{1}+\sqrt{R_{1}^{2}+X_{1}^{2}}\right]}-T_{m}}{T_{m}}$$

(7) $$K_{t}=\frac{3V_{ph}^{2}}{\omega_{s}}$$

### 2.2. Exact Model of the Induction Machine

**Figure 2:**
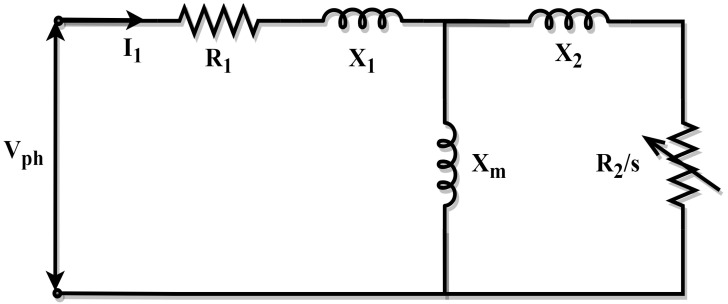
Exact model of the IM.

In contrast to the approximation circuit model, the exact circuit model considers the impacts of the rotor reactance and the magnetizing reactance in the computations. In the exact circuit model, the stator resistance ($R_{1}$), stator leakage reactance ($X_{1}$), slip (s), and rotor resistance ($R_{2}$), rotor leakage reactance ($X_{2}$), and the magnetizing reactance ($X_{m}$) are computed to determine the starting torque ($T_{st}$), maximum torque ($T_{m}$), full load power factor (pf), and full load torque ($T_{fl}$). The exact circuit model is depicted in Figure 2 [65].

The estimation problem can be stated as the following optimization problem under the assumptions of the exact circuit model:

(8) $$\mathrm{Minimize}\;f\left(x\right),\;x=\left\{R_{1},R_{2},X_{1},X_{2},s,\;X_{m}\right\}\in R^{6}$$

Subject to:

(9) $$\left.\begin{array}{c} 0\leq R_{1}\leq 1\\ 0\leq R_{2}\leq 1\\ 0\leq X_{1}\leq 1\\ 0\leq X_{2}\leq 1\\ 0\leq s\leq 1\\ 0\leq X_{m}\leq 10 \end{array}\right\}$$

where

(10) $$f\left(x\right)={f_{1}(x)}^{2}+{f_{2}(x)}^{2}+{f_{3}\left(x\right)}^{2}+{f_{4}\left(x\right)}^{2}$$

(11) f1x=KtR2sRth+R2s2+X2−TflTfl

(12) $$\;f_{2}\left(x\right)=\frac{\frac{K_{t}R_{2}}{{\left(R_{th}+R_{2}\right)}^{2}+X^{2}}-T_{st}}{T_{st}}$$

(13) $$f_{3}\left(x\right)=\frac{\frac{K_{t}}{2\left[R_{th}+\sqrt{R_{th}^{2}+X^{2}}\right]}-T_{m}}{T_{m}\left(mf\right)}$$

(14) f4x=cos⁡tan−1XRth+R2s−pfpf

(15) $$R_{th}=\frac{R_{1}X_{m}}{X_{1}+X_{m}}$$

(16) $$V_{th}=\frac{V_{ph}X_{m}}{X_{1}+X_{m}}$$

(17) $$X_{th}=\frac{X_{1}X_{m}}{X_{1}+X_{m}}$$

(18) $$K_{t}=\frac{3V_{th}^{2}}{\omega_{s}}$$

(19) $$X=X_{2}+X_{th}$$

In order to achieve the smallest possible result of Equation (8), it is also essential to satisfy an extra criterion, which is that the quantities of the calculated parameters should comply with the following constraint:

(20) $$\frac{p_{fl}-(I_{1}^{2}R_{1}+I_{2}^{2}R_{2}+P_{rot})}{p_{fl}}=\eta_{fl}$$

The rated power and rotational losses, respectively, are represented by $p_{fl}$ and $P_{rot}$ and $\eta_{fl}$ also represents the manufacturer’s efficiency. This constraint forces the computed efficiency to equal the manufacturing efficiency, ensuring that both are balanced. The variables $p_{fl}$ and $P_{rot}$ are derived, in general, using the no-load and the blocked rotor tests. These were gathered from references [18,65] to ensure consistency with other similar studies in the literature.

## 3. Proposed Algorithm

This section discusses the basic concepts of the GWO algorithm and then extends the discussion to the proposed adaptive weighted GWO algorithm.

### 3.1. Grey Wolf Algorithm

The Grey Wolf Optimizer (GWO) is a newly invented metaheuristic swarm-based algorithm that simulates the predatory behaviours of the grey wolf swarm. In the optimization, the best individual is designated as a α wolf, while the second-and third-best individuals are designated as β wolf and δ wolf, respectively, and the other agents are designated as ω wolf. Mathematically, the wolf pack’s behaviour in the prey’s vicinity is represented using Equation (21).

(21) $$X\left(t+1\right)=X_{p}\left(t\right)-A\cdot \left|C\cdot X_{p}\left(t\right)-X\left(t\right)\right|$$

where the iteration number t represents the current iteration number, X represents the wolf’s vector position, $X_{p}$ represents the prey vector position, A represents the vector coefficient, and C also represents the vector coefficients, which are represented as follows:

(22) $$A=2\cdot a\times r_{1}-a$$

(23) $$C=2\cdot a\times r_{2}$$

(24) $$a=2-\frac{2\times t}{t_{max}}$$

where $r_{1}$ and $r_{1}$ are the random numbers [0, 1], and $t_{max}$ is the maximum number of iterations. Using the top three wolves, such as α, β, and δ, the positions of the other individuals are updated, and the following mathematical model is used to calculate their positions:

(25) $$X_{a}=X_{\alpha }-A_{a}\cdot \left|C_{a}\cdot X_{\alpha }-X\right|$$

(26) $$X_{b}=X_{\beta }-A_{b}\cdot \left|C_{b}\cdot X_{\beta }-X\right|$$

(27) $$X_{c}=X_{\delta }-A_{c}\cdot \left|C_{c}\cdot X_{\delta }-X\right|$$

(28) $$X\left(t+1\right)=\frac{X_{a}\left(t\right)+X_{b}\left(t\right)+X_{c}\left(t\right)}{3}$$

where $A_{a}$, $A_{b}$, and $A_{c}$ denote wolves that are similar to A for the best three wolves, $C_{a}$, $C_{b}$, and $C_{c}$ denote wolves that are similar to C for the best three wolves and $X\left(t+1\right)$ is the individual’s final updated position.

### 3.2. Defects of Grey Wolf Optimizer

Grey Wolf Optimizer (GWO) is a popular nature-inspired optimization algorithm that models grey wolves’ social hierarchy and hunting behaviour. While GWO has been successfully applied to various optimization problems, it is important to acknowledge its limitations. The defects of the GWO are listed as follows: (i) GWO can sometimes converge prematurely to suboptimal solutions, particularly in complex optimization problems with many local optima. This is because the algorithm’s exploration and exploitation balance might not suit these problems well; (ii) in some cases, GWO may have a slow convergence rate, which could lead to increased computational time and resources. This can be particularly problematic for large-scale or real-time optimization problems; (iii) GWO is sensitive to its parameters, such as population size, the number of iterations, and various coefficients. Choosing appropriate parameter values is crucial for obtaining good results, which can be challenging, especially for non-experts; (iv) the algorithm’s search process is primarily driven by the top three wolves in the hierarchy, which can lead to a lack of diversity in the population. This may cause the algorithm to become trapped in local optima and hinder its ability to explore the entire search space effectively; (v) GWO faces scalability issues when dealing with high-dimensional optimization problems. As the number of dimensions and decision variables increases, the algorithm’s performance can degrade, making it less effective for these types of problems; (vi) GWO struggles to handle noisy optimization problems where the objective function’s evaluation is subject to noise or uncertainty. The algorithm’s performance may be affected due to its reliance on deterministic mathematical models; (vii) GWO is not well-suited for dynamic optimization problems, where the objective function or constraints change over time. The algorithm’s fixed structure and parameters might limit its ability to adapt to changing environments effectively.

Despite these limitations, GWO has shown promise in various optimization applications. To address these defects, researchers have proposed several enhancements and hybridizations of GWO with other optimization techniques, aiming to improve its performance and applicability.

### 3.3. Proposed Adaptive Weighted Grey Wolf Optimizer

It is clear from the mathematical framework in Equation (28) that the primary wolf performs a comparable position in the searching phase; each grey wolf either chases or runs away from the dominant based on the average weight of the α, β, and δ. However, even though the α is the one that is closest to the prey when the search first begins, it may be quite a distance away from the final result, especially in comparison to the beta and the delta. Therefore, at the start of the operation for searching, just the position of the alpha should be regarded in Equation (28), or its weight should be significantly higher than that of the other dominants. On the other hand, the average weight in Equation (28) goes against the idea that grey wolves live in a social hierarchy. If the social structure in the pack is strictly adhered to, the α is the leader, and they may always be the one closest to the prey. The α wolf ought to be especially significant, which indicates that the weight of the α’s position in Equation (28) should never be smaller than those of the beta and the delta positions. Therefore, the weight of the beta’s position should never be lower than that of the delta’s at any time.

In the original GWO, the vectors A and C are controlling vectors that control the exploration and exploitation abilities. Both vectors are controlled by the adaptive parameter called a. The value of a linearly decreases from 2 to 0 during the iterative process, as discussed in Equation (24). Since the parameter *a* indirectly controls the exploration and exploitation of the GWO, it is suggested to modify the expression so that the performance of the original GWO is increased further. Therefore, in this study, the modified expression for the vector a is proposed, and it is provided in Equation (29).

(29) $$a=2-\left(\left(\frac{t}{t_{max}}\right)\times \cos\left(rand\right)\right)$$

where rand denotes the uniform random number between [0, 1], t denotes the current iteration, and $t_{max}$ denotes the maximum number of iterations. The modification due to Equation (29) allows a to follow a nonlinear decreasing trend, ensuring that exploration is more active in the early iterations while transitioning smoothly into exploitation in later stages. The introduction of the cosine term adds an element of randomness, making it less likely for the algorithm to be trapped in local optima.

Additionally, adaptiveness and weights are also introduced in the original GWO algorithm. When the hunt starts, the α is the closest one, and the others are insignificant. Therefore, their position ought to be contributed to the individuals seeking, but the other candidates may be neglected. The value of 0 indicates that the weight of the α ought to be close to 1 at the start, although the corresponding weights of the β and δ variables can be close to 0 at this point. According to Equation (28), the final state of the pack should consist of the α, β, and δ wolves encircling the prey. This indicates that all three packs have the same amount of weight. Along with the searching technique from the start to the end, the β shows up with the α because it always ranks second, and then δ comes up with the β. After all, it ranks third. When 0 is present, it indicates that the weights of the β and δ variables emerge simultaneously with the cumulative iteration number. As a result, the weight of the α should be decreased, while the weights of the β and δ should emerge. There should be some variation when each weight is added together, but the total should not exceed one. After that, Equation (28) is revised as follows:

(30) $$X\left(t+1\right)=\sigma_{1}X_{a}\left(t\right)+\sigma_{2}X_{b}\left(t\right)+{\sigma_{3}X}_{c}\left(t\right)$$

(31) $$\sigma_{1}+\sigma_{2}+\sigma_{3}=1$$

where $\sigma_{1}$, $\sigma_{2}$, and $\sigma_{3}$ are the weight factors of the respective wolves. The weights $\sigma_{1}$, $\sigma_{2}$, and $\sigma_{3}$ should always satisfy the condition $\sigma_{1}\geq \sigma_{2}\geq \sigma_{3}$. Along with the search operation, the value of $\sigma_{1}$ reduced from one to 0.333; simultaneously, $\sigma_{2}$ and $\sigma_{3}$ would be increased from 0 to 0.333. When we limit the range of an angle θ to be between 0 and arccos(0.333), we can use a cosine function to represent $\sigma_{1}$. In general, this can be performed by introducing a cosine function. As discussed earlier, all the weights must be adaptive during the iteration process. This study introduces an arctangent function so that the value varies from 0 to π/2. Therefore, another parameter is also introduced in this study, as presented in Equation (32), and the expression for θ is provided in Equation (33).

(32) $$\varphi =0.5\times \arctan\left(t\right)$$

(33) $$\theta =\frac{2}{\pi }\times \arccos\left(0.33\right)\times \arctan\left(t\right)$$

Finally, all the weights, such as $\sigma_{1}$, $\sigma_{2}$, and $\sigma_{3}$ are formulated and shown in Equations (34)–(36), proving that the weights are adaptive, which influences the performance of the original GWO algorithm.

(34) $$\sigma_{1}=\cos\theta$$

(35) $$\sigma_{2}=0.5\times \sin\theta \times \cos\varphi$$

(36) $$\sigma_{3}=1-\sigma_{1}-\sigma_{2}$$

**Figure 3:**
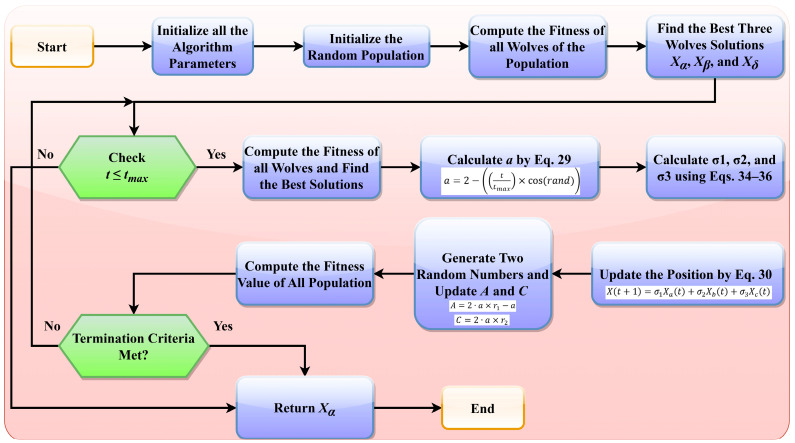
Flowchart of the proposed AWGWO algorithm.

The adaptive weighting mechanism proposed in this study ensures that α, β, and δ wolves dynamically adjust their contributions, allowing the algorithm to transition between exploration and exploitation phases efficiently. The proposed adaptive weight mechanism significantly enhances GWO as follows: (i) since $\sigma_{1}$ is large initially, the alpha wolf has dominant influence, ensuring broad exploration; (ii) as iterations progress, $\sigma_{1}$ decreases while $\sigma_{2}$ and $\sigma_{3}$ increase, allowing beta and delta wolves to contribute more to fine-tuning the solution; (iii) the adaptive weights prevent premature convergence and allow AWGWO to escape local optima more effectively. This study, therefore, can demonstrate that the variable weights are consistent with the theory, which is the social structure of the functions that the grey wolves exhibit when searching. The adaptive equations of the controlling parameter are developed in this study, and as a result, it is adjusted so that it decreases exponentially from its highest value. This is performed to limit the likelihood of being stuck in the locally optimal solution. The pseudocode of the proposed AWGWO algorithm is shown in Algorithm 1. The flowchart of the proposed AWGWO is shown in Figure 3.
**Algorithm 1:** Pseudocode of the proposed AWGWO**1:** **2:** **3:** **4:** **5:** **6:** **7:** **8:** **9:** **10:** **11:** **12:** **13:** **14:**Initialize the $t_{max}$ and population size N Initialize the random population and random population solution Initialize the GWO parameters, such as a, A, and C Identify the best three individuals, such as $X_{\alpha }$, $X_{\beta }$, and $X_{\delta }$ ** For $i=1:\;t_{max}$ do**  **For i=1:N do**    The position of the current search agent is updated using Equation (30)  **End For** Update the value of a using Equation (29) and A and C using Equations (22) and (23) Find the fitness of all the search agents Position of $X_{\alpha }$, $X_{\beta }$, and $X_{\delta }$ is updated using Equations (25)–(27) t=t+1 **End For** **Return** the best solution 

### 3.4. Computational Complexity

The complexity of the original GWO is determined per iteration: (i) fitness evaluation for each population, (ii) position updates based on the three best wolves, and (iii) controlling the balance between exploration and exploitation. Given a population size of N, problem dimensionality dim, and maximum iterations $t_{max}$, the position update operation has a complexity of $O\left(N\cdot dim\right)$ per iteration. The overall complexity of GWO is $O\left(t_{max}\cdot N\cdot dim\right)$. For the proposed AWGWO, additional computational overhead arises due to the adaptive weighting mechanism. The adaptive mechanism involves dynamically adjusting weight factors ($\sigma_{1},\sigma_{2},\sigma_{3}$) using trigonometric and arctangent functions, along with modifying the exploration-–exploitation balance. These weight calculations introduce additional $O\left(N\cdot \log t_{max}\right)$ complexity per iteration. The overall complexity of AWGWO increases to $O\left(t_{max}\cdot N\cdot \left(dim+\log t_{max}\right)\right)$. While the added logarithmic term slightly increases computational cost, it enhances convergence behaviour, leading to improved search efficiency and solution accuracy compared to standard GWO.

## 4. Results and Discussions

This section of the paper comprehensively discusses the performance of the proposed AWGWO and other selected algorithms for numerical and real-world engineering design problems.

### 4.1. Results Obtained for Benchmark Problems

To validate the performance of the proposed algorithm, 10 traditional benchmark problems are considered in this study. The selected 10 test problems have both unimodal and multi-modal abilities with fixed and variable dimensions. The performance of the AWGWO is compared with the PSO, DE, GSA, GWO, IGWO-DS, LSGWO, and GWO-CM. Each algorithm is executed 30 times individually to have a fair comparison. The parameter settings of all selected algorithms under consideration were derived from the base article and trial and error. The population size is 30, and the maximum number of iterations is selected as 500 for the numerical problems. The characteristics of the selected benchmark functions are detailed in Table 1. These functions were carefully chosen to provide a comprehensive analysis of the AWGWO algorithm’s exploration and exploitation capabilities across varying levels of complexity. Specifically, functions TF1 through to TF4 exhibit unimodal characteristics, with 30 problem dimensions, making them ideal for testing the algorithm’s ability to exploit search spaces to find global optima efficiently. In contrast, functions TF5 to TF7 feature multimodal characteristics with 30 dimensions, posing a challenge by introducing multiple local optima, which require effective exploration to avoid premature convergence. Lastly, functions TF8 through to TF10 are also multimodal but with significantly lower dimensions, adding another layer of difficulty by requiring the algorithm to balance its exploration and exploitation strategies in a reduced search space. The detailed statistical results, including the minimum (Min), mean (Mean), maximum (Max), standard deviation (STD) of the objective function values, run-time (RT), and Friedman’s Ranking Test (FRT) values, were computed for each algorithm under study and are presented in Table 2.

The performance of the AWGWO algorithm, as evaluated on unimodal functions (TF1–TF4), demonstrates its ability to effectively exploit search spaces with high precision. The improvement in exploitation capability can be attributed to the incorporation of adaptive weight factors, which enhance the original GWO. These weight factors adjust dynamically, enabling AWGWO to maintain a more refined search in the solution space. The adaptive mechanisms also allow the algorithm to explore regions near previously discovered solutions, improving its ability to locate global optima more reliably. Consequently, the modifications made to the GWO framework have significantly enhanced its performance on unimodal test cases. When applied to multimodal functions, the AWGWO algorithm’s exploration capabilities become even more apparent. As shown in Table 2, AWGWO excels in exploring highly competitive solutions across all multimodal test cases, outperforming all other algorithms. This is particularly evident in the results of TF5-TF8, where the objective problems involve several local optima. The adaptive weight factors enable AWGWO to maintain a fine balance between global and local searches. The statistical results further support this, with AWGWO showing superior performance compared to GWO and other algorithms in terms of accuracy, as measured by the STD index. AWGWO consistently produces more stable and precise solutions, particularly in complex multimodal test cases. The enhanced exploration mechanism, driven by the adaptive weight factors, gives AWGWO a clear advantage in handling these challenging optimization problems. In terms of computational complexity, the RT of the proposed AWGWO algorithm was also assessed and recorded in Table 2. Although the RT of the original GWO is slightly lower, the increase in RT for AWGWO is marginal and is primarily due to the introduction of the adaptive weight mechanism. The adaptive mechanisms enhance the algorithm’s overall performance without introducing excessive computational overhead, making it a highly efficient and practical solution for both unimodal and multimodal optimization problems. The best values are highlighted in bold in all tables.

**Figure 4:**
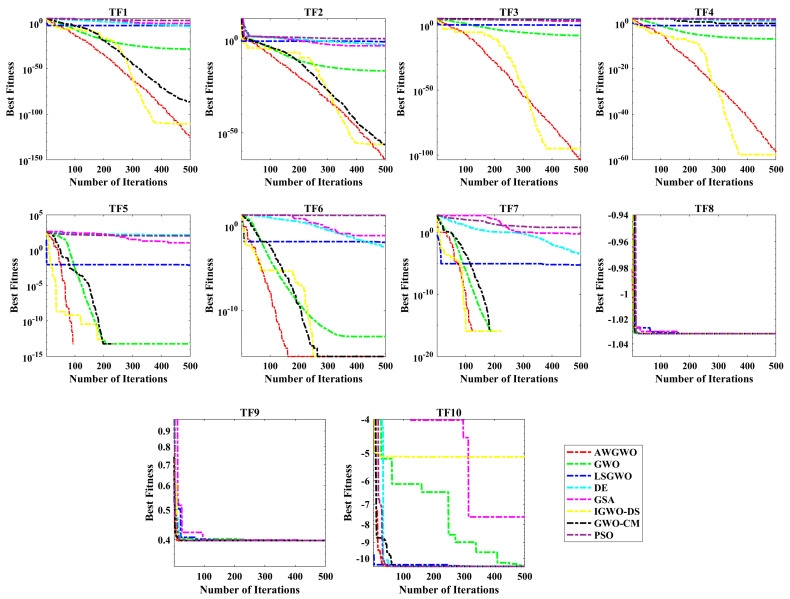
Convergence curves of all algorithms for numerical functions.

Table 2 also records the FRT values and mean FRT values. Based on mean FRT values, the proposed AWGWO ranked first, followed by IGWO-DS, DE, GWO, LSGWO, GWO-CM, PSO, and GSA. Figure 4 presents the convergence characteristics of all selected algorithms. Each of the algorithms demonstrates competitive performance, consistently delivering superior results. Notably, Figure 4 provides insights into the convergence timeframe, highlighting the stages at which AWGWO begins to outperform the GWO. It is evident from the figure that as the iterations progress, AWGWO achieves significantly better outcomes, converging toward highly accurate solutions that closely approximate the global optima. One key observation from Figure 4 is that AWGWO benefits from an extended number of iterations, which allows it to refine its search process and converge on more precise solutions. The algorithm’s ability to maintain a balance between exploration and exploitation is evident in its ability to highlight local search and fine-tuning during the latter stages of the optimization process. The comparison between the convergence curves of AWGWO and other algorithms highlights this improvement, with AWGWO demonstrating more rapid and efficient convergence. Moreover, the effectiveness of AWGWO in improving the fitness of all wolves is evident from the convergence plots. By ensuring that all agents contribute to the search in an optimized manner, AWGWO increases the likelihood of finding improved solutions. The adaptive mechanisms embedded within AWGWO enable it to dynamically adjust its search strategies, resulting in enhanced convergence.

**Figure 5:**
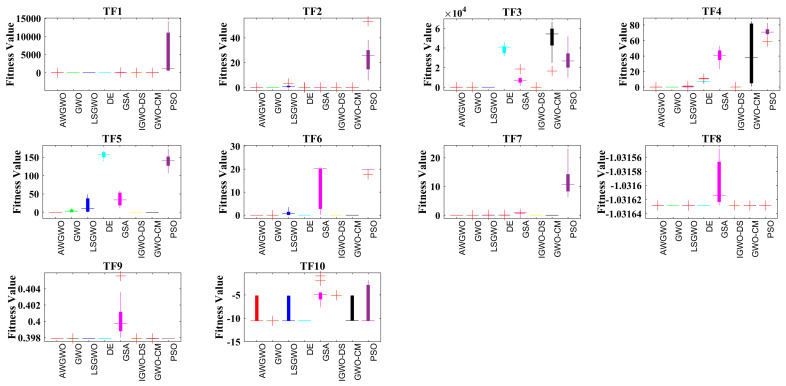
Boxplots of all algorithms for numerical functions.

In addition to the convergence analysis, Figure 5 provides a detailed visualization of the stability of the selected algorithms through boxplots. From Figure 5, it is clear that AWGWO outperforms all other algorithms in terms of stability. The robustness of AWGWO is further emphasized by its ability to handle both unimodal and multimodal functions with equal efficiency. While many algorithms struggle to maintain stability in the face of complex optimization challenges, AWGWO’s design enables it to adapt its search dynamics, ensuring reliable and consistent results. This makes AWGWO not only an efficient optimizer in terms of convergence speed, but also a highly stable one, capable of delivering reliable solutions over a range of test cases.

### 4.2. Results Obtained for Parameter Estimation Problem

It is demonstrated in this paper that the proposed AWGWO can be utilized to identify the best parameters of one induction motor when the approximate circuit model and exact circuit model are considered. Technical specifications of the motor utilized in the studies are presented in Table 3. In the paper, the study employed the AWGWO model to estimate the induction motor parameters, and the results are presented. The performance comparison is made among various metaheuristic algorithms, such as PSO, DE, GSA, and GWO. The parameter settings of all selected algorithms under consideration were derived from the base article and trial and error. The population size was 40, and the maximum number of iterations was selected as 1000. Table 3 lists the nameplate details of IM under study.

The simulation results are presented in two sub-sections. The first sub-section uses an approximate circuit model to describe the results obtained by the AWGWO, GWO, PSO, DE, and GSA. The second sub-section uses an exact circuit model to describe the results obtained by the AWGWO, GWO, PSO, DE, and GSA. In addition, the proposed algorithm is also compared with other improved variants of GWO algorithms, such as IGWO-DS, LSGWO, and GWO-CM, for both case studies.

#### 4.2.1. Results on Approximate Circuit Model

The population size *N* and the maximum number of iterations $t_{max}$ have the most influence on the performance expectations of all selected algorithms. As stated earlier, the population and the maximum number of iterations are selected as 40 and 1000, respectively. The behaviour of the AWGWO over the motor parameter identification problem is examined in this study and compared with four other algorithms, such as PSO, DE, GSA, and GWO. Firstly, the approximate circuit model simulation starts with PSO, DE, GSA, GWO, and AWGWO algorithms and extends to the different variants, such as AWGWO, IGWO-DS, LSGWO, and GWO-CM. Table 4 lists all the unknown parameters of the approximate circuit model. It also displays the obtained fitness function values. It is observed that the solution accuracy of the proposed algorithm to the approximate circuit model is increased. This is due to the adopted adaptive weight update mechanism and adaptive parameter that controls the exploration and exploitation of the AWGWO algorithm.

Table 4 shows that the fitness values obtained by the proposed AWGWO algorithm are better than the original GWO algorithm. In addition, it is also much better than other selected algorithms. At the same time, the prediction of performance parameters of the induction motor, such as full-load torque, maximum torque, and starting torque, based on the computed unknown variables is listed in Table 5. The error between the predicted torques and experimental torques is also provided. Based on the observation from Table 5, the proposed AWGWO algorithm gave the best results of all selected algorithms. Based on the results obtained, the ranking of the algorithms is assigned. Based on the fitness values, the proposed algorithm ranked first, followed by DE, GWO, GSA, and PSO.

In addition, the statistical analysis is also carried out by considering the results obtained by all the selected algorithms. The statistical parameters, such as Min, Max, Average, STD, and RT, are recorded in Table 6. It is visible from Table 6 that the statistical results are better for the proposed algorithm. Especially the average and STD values obtained with the proposed algorithm are better than all other selected algorithms. In addition, the RT values are listed. It is quite obvious that the RT value increases when the adaptive mechanism is included. Though the performance of the proposed algorithm is better, the RT of the proposed algorithm is high, and this is due to the fact that the adaptive weight mechanism took extra RT since the weights are updated after every single iteration. However, the performance of the proposed algorithm is excellent, and it can be considered as a reliable tool for the parameter estimation of the IMs.

**Figure 6:**
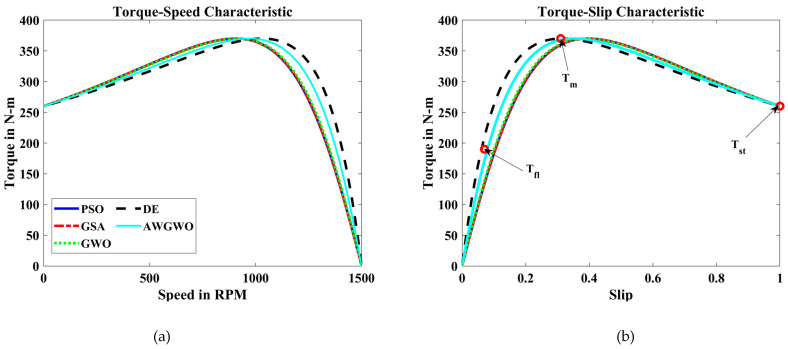
Characteristics of IM (approximate circuit model) for different algorithms; (**a**) torque–speed characteristics, (**b**) torque–slip characteristics.

In order to visualize the characteristic performance curves of the IM by considering the approximate circuit model, the torque–speed and torque–slip characteristics are plotted based on the computed unknown parameters of the IM, as shown in Figure 6. To observe the impact of all selected algorithms, the curves are plotted for algorithms. The algorithms, such as PSO, GSA, and GWO, cannot find the best optimal solution, and this statement can be proved by visualizing the plots. The performance of DE is almost comparable to the proposed algorithm. However, the reliability of the proposed algorithm is better than any other algorithm. In addition, to observe the convergence rate of the GWO algorithm, the convergence curves are plotted and displayed in Figure 6.

**Figure 7:**
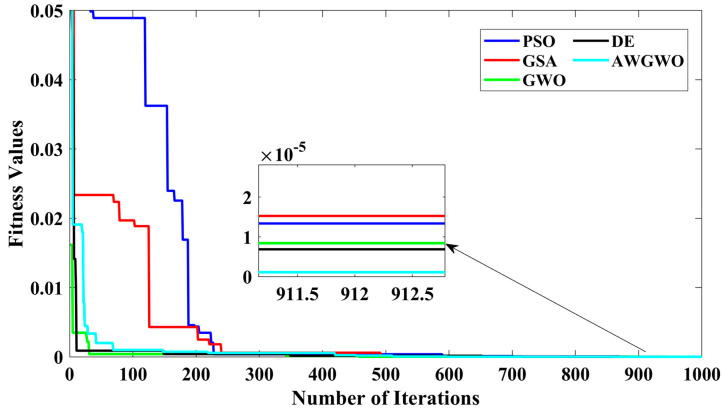
Convergence curves of all algorithms (approximate circuit model).

From Figure 7, it is clearly visible that the convergence speed of the proposed algorithm is higher than any other algorithm. Though the performance of the DE algorithm is comparable with the proposed algorithm, the convergence speed is poorer than the AWGWO. Therefore, it concluded from Figure 6 that the convergence rate is better when the proposed AWGWO is applied to this model. However, it is important to notice that this statement may not be true for other applications.

In order to compare the performance of the proposed AWGWO, it is also compared with other variants of GWO, such as LSGWO, IGWO-DS, and GWO-CM. Table 7 lists all the unknown parameters of the approximate circuit model by all selected variants of the GWO algorithm. The obtained fitness function values for each algorithm are displayed. It is obvious that the solution accuracy of the proposed algorithm to the IMs parameter estimation problem is increased. Table 7 shows that the fitness values obtained by the proposed AWGWO algorithm are lower and much better than all other selected variants of the GWO algorithm. At the same time, the prediction of performance parameters of the induction motor, such as full-load torque, maximum torque, and starting torque, based on the computed unknown variables is listed in Table 8. The error between the predicted torques and experimental torques is also provided. Based on the observation from Table 8, the proposed AWGWO algorithm gave the best results, similar to the previous discussions. It is also confirmed that the torque error produced by the AWGWO algorithm is lesser than LSGWO, IGWO-DS, and GWO-CM.

In addition, the statistical analysis is also carried out by considering the results obtained by the different maximum iteration counts. It is visible from Table 9 that the statistical result of the proposed algorithm is better than other variants of the GWO algorithm. Especially, the average and STD values obtained by the proposed algorithm show better results among all other GWO variants. In addition, the RT values are listed. Obviously, the RT value of all GWO versions is higher than the basic GWO. However, the RT values of IGWO-DS and GWO-CM are much higher than the LSGWO and AWGWO algorithms. The RT value of the AWGWO is much better than any other considered variants; however, the RT value of LSGWO is almost equal to the AWGWO, with the performance less than the proposed AWGWO algorithm.

**Figure 8:**
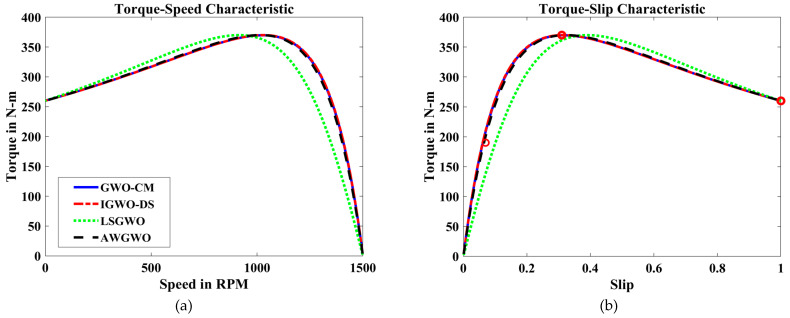
Characteristics of IM (approximate circuit model) for different GWO variants; (**a**) torque–speed characteristics, (**b**) torque–slip characteristics.

**Figure 9:**
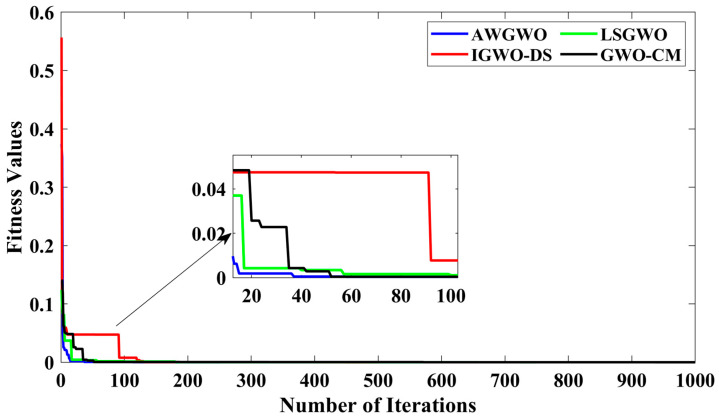
Convergence curves for different GWO variants (approximate circuit model).

In order to visualize the characteristic performance curves of the IM obtained by all variants of GWO algorithms by considering the approximate circuit model, the torque–speed and torque–slip characteristics are plotted based on the computed unknown parameters of the IM, as shown in Figure 8. To observe the impact of different variants of GWO algorithms, the curves are plotted. The proposed AWGWO and IGWO-DS algorithms could be able to find the best optimal solution, and this statement can be proved by visualizing the plots. In addition, to observe the convergence rate of the variants of the GWO algorithm, the convergence curves are plotted and displayed in Figure 9. From Figure 9, it is clearly visible that the convergence rate of the proposed AWGWO and IGWO-DS is higher than the LSGWO and GWO-CM. Therefore, it is concluded from Figure 9 that the convergence rate is better when we select the proposed algorithm for the equivalent circuit parameter estimation of the IMs.

#### 4.2.2. Results for Exact Circuit Model

This case study aims to test the algorithm performance when the number of design parameters, i.e., problem dimension, increases. Similar to the previous test case, the population and the maximum number of iterations are 40 and 1000, respectively. The behaviour of the AWGWO over the motor parameter identification of the exact circuit model is examined in this study and compared with four other algorithms, such as PSO, DE, GSA, and GWO. Firstly, the exact circuit model simulation starts with PSO, DE, GSA, GWO, and AWGWO algorithms and extends to the different variants, such as AWGWO, IGWO-DS, LSGWO, and GWO-CM. Table 10 lists all the unknown parameters of the exact circuit model. It also displayed the obtained fitness function values. It is observed that the solution accuracy of the proposed algorithm to the exact circuit model is increased. This is due to the adopted adaptive weight update mechanism and adaptive parameter that controls the exploration and exploitation of the AWGWO algorithm.

Table 10 shows that the fitness values obtained by the proposed AWGWO algorithm are better than the original GWO algorithm. In addition, it is also much better than other selected algorithms. At the same time, the prediction of performance parameters of the induction motor, such as full-load torque, maximum torque, and starting torque, based on the computed unknown variables is listed in Table 11. The error between the predicted torques and experimental torques is also provided. Based on the observation from Table 11, the proposed AWGWO algorithm gave the best results of all selected algorithms. Based on the results obtained, the ranking of the algorithms is assigned. Based on the fitness values, the proposed algorithm stood first, followed by DE, GWO, GSA, and PSO.

In addition, the statistical analysis is also carried out by considering the results obtained by all the selected algorithms. The statistical parameters, such as Min, Max, Average, STD, and RT, are recorded in Table 12. It is visible from Table 12 that the statistical results are better for the proposed algorithm. In particular, the average and STD values obtained with the proposed algorithm are better than all other selected algorithms. In addition, the RT values are listed. It is quite obvious that the RT value increases when the adaptive mechanism is included. Though the performance of the proposed algorithm is better, the RT of the proposed algorithm is high, and this is due to the fact that the adaptive weight mechanism took extra RT since the weights are updated after every single iteration. However, the performance of the proposed algorithm is excellent, and it can be considered as a reliable tool for the parameter estimation of the IMs.

**Figure 10:**
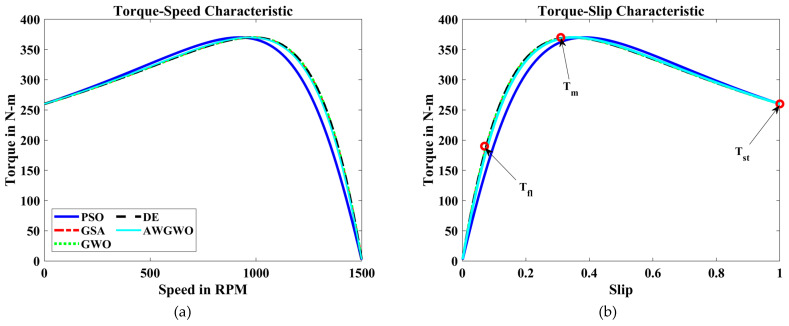
Characteristics of IM (exact circuit model) for different algorithms; (**a**) torque–speed characteristics, (**b**) torque–slip characteristics.

**Figure 11:**
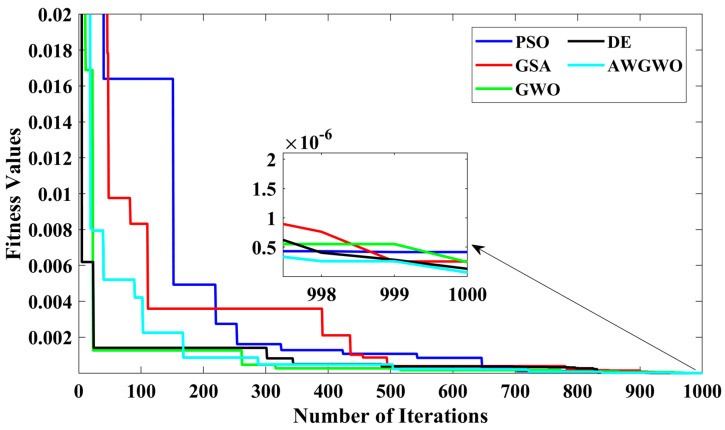
Convergence curves for different algorithms (exact circuit model).

In order to visualize the characteristic performance curves of the IM by considering the exact circuit model, the torque–speed and torque–slip characteristics are plotted based on the computed unknown parameters of the IM, as shown in Figure 10. To observe the impact of all selected algorithms, the curves are plotted for algorithms. The algorithms, such as PSO, GSA, and GWO, cannot find the best optimal solution, and this statement can be proved by visualizing the plots. The performance of DE is almost comparable to the proposed algorithm. However, the reliability of the proposed algorithm is better than any other algorithm. In addition, to observe the convergence rate of the AWGWO algorithm, the convergence curves are plotted and displayed in Figure 11.

The results shown in Figure 11 highlight the advantage of the proposed AWGWO algorithm in terms of convergence speed. While the DE algorithm demonstrated competitive performance in terms of solution quality, it lagged behind AWGWO when it came to convergence efficiency. The faster convergence of AWGWO indicates its ability to rapidly explore and exploit the solution space, reducing the time required to reach optimal or near-optimal solutions. This is particularly beneficial in real-world applications where computational efficiency is crucial. Additionally, the ability of AWGWO to avoid premature convergence and effectively navigate multimodal error landscapes plays a key role in achieving this superior convergence speed. The adaptive mechanism incorporated within AWGWO enables it to adjust its exploration and exploitation balance dynamically, allowing it to maintain diversity in the search process while improving on optimal solutions more efficiently. This makes AWGWO a highly suitable option for optimization tasks where time constraints and solution precision are of paramount importance. Consequently, Figure 11 emphasizes that the proposed AWGWO not only outperforms other algorithms in terms of solution accuracy but also excels in computational efficiency.

In order to compare the performance of the proposed AWGWO, it is also compared with other variants of GWO, such as LSGWO, IGWO-DS, and GWO-CM. Table 13 lists all the unknown parameters of the exact circuit model by all selected variants of the GWO algorithm. The obtained fitness function values for each algorithm are displayed. It is obvious that the solution accuracy of the proposed algorithm to the IMs parameter estimation problem is increased. Table 13 shows that the fitness values obtained by the proposed AWGWO algorithm are less and much better than all other selected variants of the GWO algorithm. At the same time, the prediction of performance parameters of the induction motor, such as full-load torque, maximum torque, and starting torque, based on the computed unknown variables is listed in Table 14. The error between the predicted torques and experimental torques is also provided. Based on the observation from Table 13, the proposed AWGWO algorithm gave the best results, similar to the previous discussions. It is also confirmed that the torque error produced by the AWGWO algorithm is lesser than LSGWO, IGWO-DS, and GWO-CM.

In addition, the statistical analysis is also carried out by considering the results obtained by the different maximum iteration counts. It is visible from Table 15 that the statistical result of the proposed algorithm is better than other variants of the GWO algorithm. In particular, the average and STD values obtained by the proposed algorithm show better results among all other GWO variants. In addition, the RT values are listed. Obviously, the RT value of all GWO versions is higher than the basic GWO. However, the RT values of IGWO-DS and GWO-CM are much higher than the LSGWO and AWGWO algorithms. The RT value of the AWGWO is much better than any other considered variants; however, the RT value of LSGWO is almost equal to the AWGWO, with the performance lesser than the proposed AWGWO algorithm.

**Figure 12:**
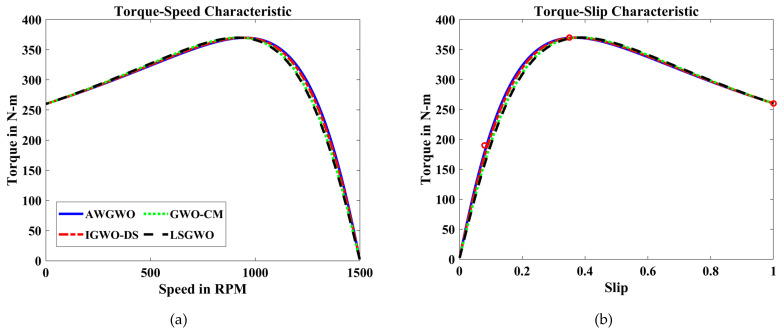
Characteristics of IM (exact circuit model) for different variants of GWO; (**a**) torque–speed characteristics, (**b**) torque–slip characteristics.

To further illustrate the characteristic performance curves of the IM obtained using various GWO algorithm variants, the torque–speed and torque–slip characteristics are plotted based on the estimated unknown parameters of the IM, as shown in Figure 12. These plots provide a clear visualization of how different GWO algorithm variants perform in terms of parameter estimation and their impact on motor performance. By comparing these curves, it becomes evident that the proposed AWGWO and IGWO-DS algorithms are able to find the most optimal solutions, resulting in smooth and consistent torque–speed and torque–slip characteristics. The superiority of these two algorithms can be observed through their ability to more accurately model the IM’s behaviour, as reflected in the sharper convergence towards the ideal performance. The analysis suggests that AWGWO and IGWO-DS have a higher degree of precision in estimating the parameters, which directly influences the motor’s performance characteristics. The plots further validate that these algorithms are more adept at handling the complex, nonlinear behaviour of IMs, especially under varying load conditions, making them the most reliable choices among the GWO variants tested.

**Figure 13:**
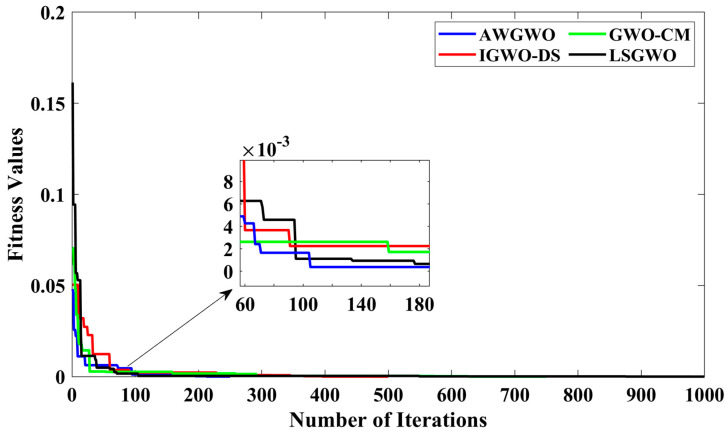
Convergence curves for different variants of GWO (exact circuit mode4.3. Results Obtained for Commercial Induction Motors.

Furthermore, to thoroughly examine the convergence rates of the various GWO algorithm variants, the convergence curves are plotted and displayed in Figure 13. From Figure 13, it is evident that the convergence rates of the proposed AWGWO and IGWO-DS algorithms are significantly higher compared to the LSGWO and GWO-CM algorithms. The sharper decline in the convergence curves of AWGWO and IGWO-DS demonstrates their superior efficiency in reaching optimal solutions faster, with fewer iterations required to achieve convergence. The enhanced performance can be attributed to the adaptive mechanisms and improved search strategies incorporated in AWGWO, which allow for better exploration of the search space and avoidance of premature convergence to sub-optimal solutions. Consequently, it can be concluded from Figure 13 that these algorithms offer a distinct advantage in terms of convergence speed when applied to the equivalent circuit parameter estimation of IMs, making them more suitable for time-sensitive and computationally demanding applications.

To further analyze the performance of the proposed algorithm, eight commercial induction motors are considered. Motor 1 is from Hindustan Electric Motors, Motor 2 to Motor 4 are from Amber Engineering Enterprise, Motor 5 to Motor 7 are from ABB, and Motor 8 is from WEG. The specifications of all eight motors are listed in Table 16. For this experimental study, an exact equivalent circuit model is considered. The proposed AWGWO, PSO, GSA, GWO, and DE are considered for this analysis. Similarly to the previous case study, the population size and the maximum number of iterations are selected as 40 and 1000. All algorithms are executed 30 times individually for a fair experimental comparison. All the obtained parameters of all commercial motors are recorded in Table 17, and Table 17 also shows that the best fitness values obtained by the proposed AWGWO algorithm are better than the original GWO algorithm and other selected algorithms for all commercial motors. The best fitness obtained by the proposed algorithm is significantly better than any of the selected algorithms.

Similar to previous discussions, the prediction of performance parameters of all eight commercial induction motors, such as full-load torque, maximum torque, and starting torque, based on the computed unknown variables listed in Table 18. The error between the predicted torques and experimental torques is also provided. Based on the observation from Table 18, the proposed AWGWO algorithm gave the best results of all selected algorithms for most of the selected commercial motors. Based on the results obtained, the ranking of the algorithms was assigned. Based on the fitness values, the proposed algorithm ranked first, followed by DE, GWO, GSA, and PSO. In addition, the statistical analysis was also carried out by considering the results obtained by all the selected algorithms for all eight commercial motors. The statistical parameters, such as Min, Max, Average, STD, and RT, are recorded in Table 19. It is visible from Table 19 that the statistical results are better for the proposed algorithm. Especially the average and STD values obtained with the proposed algorithm are better than all other selected algorithms. In addition, the RT values are listed. It is quite obvious that the RT value increases more than the original GWO algorithm when the adaptive mechanism is included. Though the performance of the proposed algorithm is better, the RT of the proposed algorithm is high, and this is due to the fact that the adaptive weight mechanism took extra RT since the weights are updated after every single iteration. However, the performance of the proposed algorithm is excellent, and it can be considered as a reliable tool for the parameter estimation of commercial IMs.

**Figure 14:**
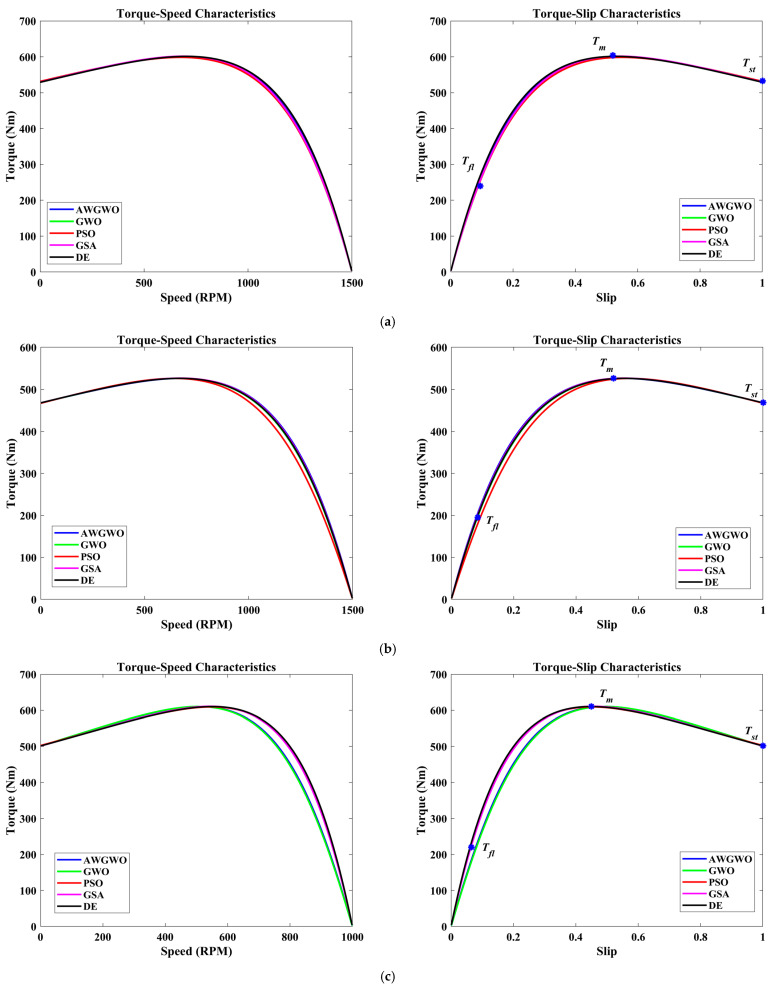

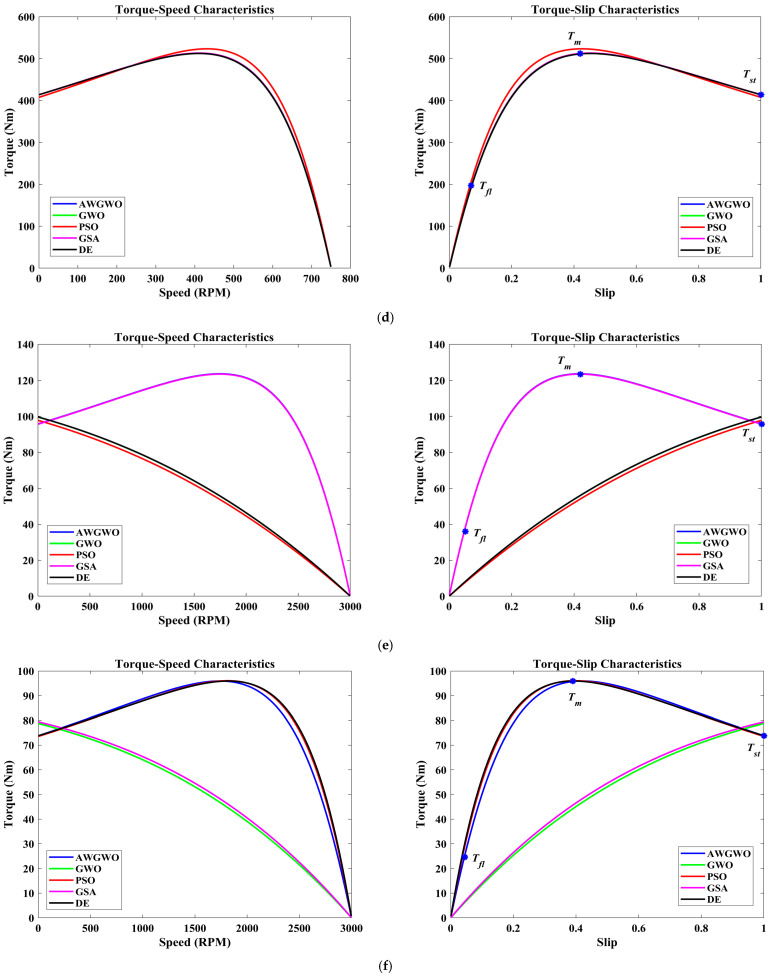

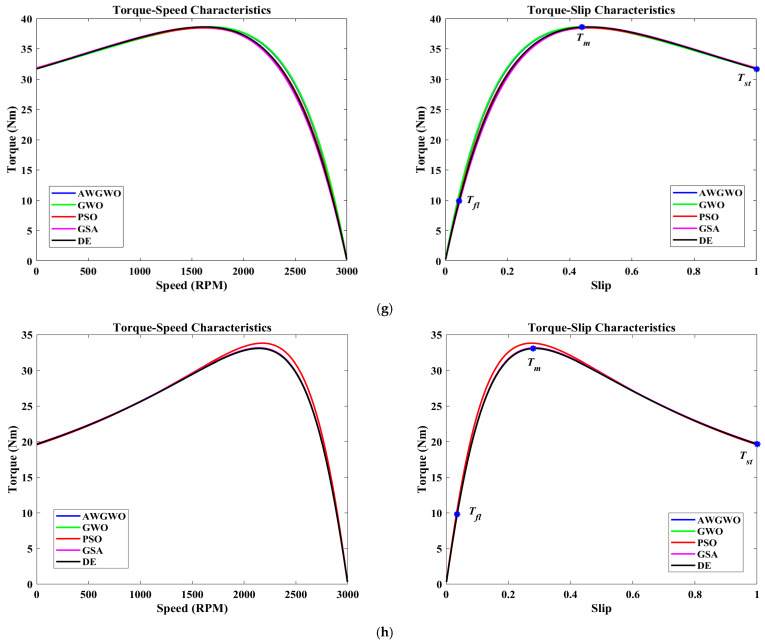
Torque–speed and torque–slip characteristics of commercial motors: (**a**) Motor 1, (**b**) Motor 2, (**c**) Motor 3, (**d**) Motor 4, (**e**) Motor 5, (**f**) Motor 6, (**g**) Motor 7, (**h**) Motor 8.

**Figure 15:**
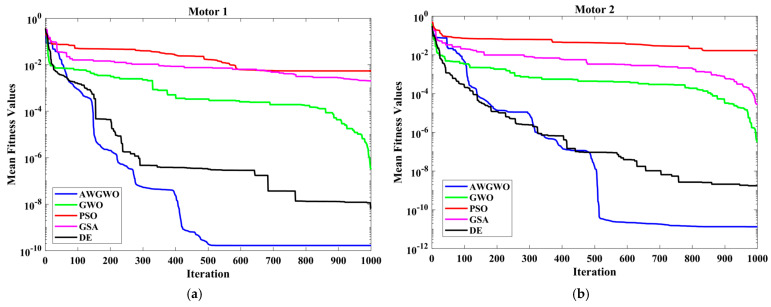

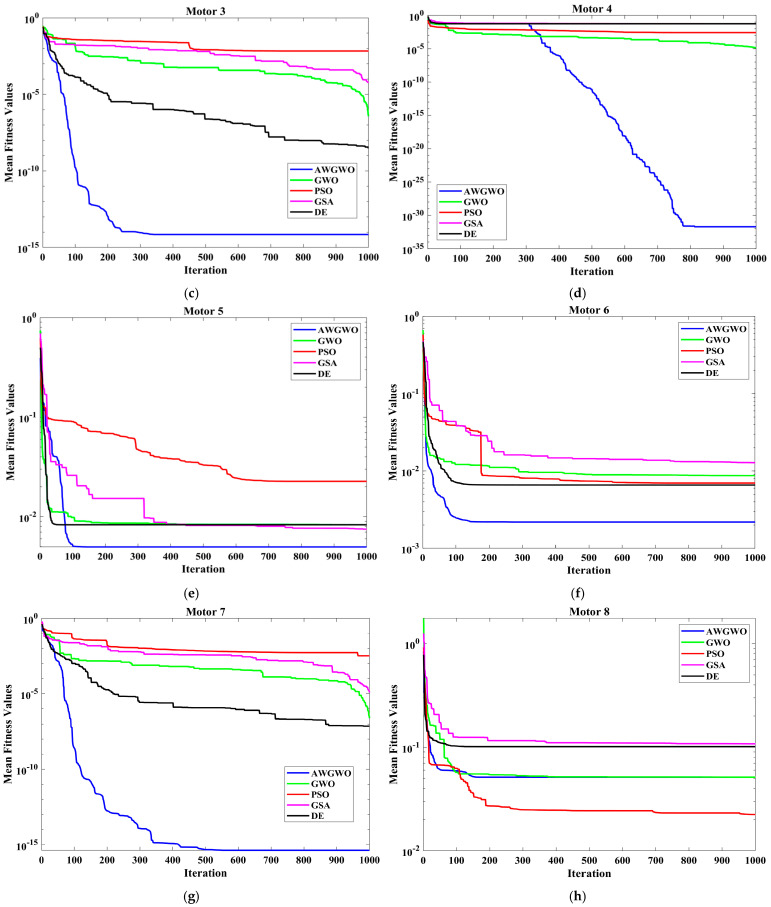
Convergence curves of all algorithms for commercial motor models: (**a**) Motor 1, (**b**) Motor 2, (**c**) Motor 3, (**d**) Motor 4, (**e**) Motor 5, (**f**) Motor 6, (**g**) Motor 7, (**h**) Motor 8.

In order to visualize the characteristic performance curves of the commercial IMs by considering the exact circuit model, the torque–speed and torque–slip characteristics are plotted based on the computed unknown parameters of the IM listed in Table 17, as shown in Figure 14. To observe the impact of all selected algorithms, the curves are plotted for algorithms. In most of the cases, the traditional PSO algorithm failed to predict the proper operating characteristics. In a few cases, the traditional GWO and DE also failed to extract the accurate parameters. The same resulted in the operating characteristics. For most of the cases, the proposed AWGWO algorithm could find the optimal parameters of the commercial motors. The algorithms, such as PSO, DE, and GWO, cannot find the best optimal solution, and this statement can be proved by visualizing the plots. The performance of GSA is acceptable but poorer than the proposed algorithm. In all the characteristic curves, the full-load torque, maximum torque, and starting torque are also marked. These torque values match with the torques specified in the datasheet. The performance of the basic algorithms is poor, and this is due to the poor exploration and exploitation ability within the specified bounds. The reliability of the proposed algorithm is better than any other algorithm, and the same was evidenced by referring to the STD values recorded in Table 19. In addition, to observe the convergence rate of the AWGWO algorithm and other algorithms, the convergence curves for all commercial motors are also plotted and displayed in Figure 15. From Figure 15, it is clearly visible that the convergence speed of the proposed algorithm is higher than any other algorithm. Though the performance of the DE and GSA is comparable with the AWGWO, the convergence speed is poorer than the AWGWO.

Comparing the AWGWO algorithm with other algorithms such as PSO, DE, GSA, GWO, GWO-DS, LSGWO, and GWO-CM, it is essential to highlight the limitations of the compared algorithms. The primary disadvantage of traditional algorithms, such as PSO, DE, and GSA, is premature convergence, particularly when solving complex multimodal problems and IM parameter estimation problems. Additionally, GWO and its variants, such as GWO-DS, LSGWO, and GWO-CM, frequently struggle with balancing exploration and exploitation, leading to suboptimal results in parameter estimation for IMs. The AWGWO algorithm overcomes these limitations by introducing an adaptive weight mechanism, which dynamically adjusts the search agents’ influence during the optimization process, allowing the algorithm to maintain a better balance between exploration and exploitation. The adaptability significantly enhances the global search capability, mitigates premature convergence, and improves stability, particularly in high-dimensional optimization problems. Later, the AWGWO demonstrates superior performance by avoiding the drawbacks of traditional algorithms, making it more effective for real-world parameter estimation problems.

In summary, AWGWO’s adaptive features significantly enhance the exploration and exploitation balance, leading to superior performance across both numerical functions and real-world parameter estimation problems. Its increased computational complexity is minimal compared to the gains in optimization accuracy and stability, making it a reliable tool for solving complex real-world optimization problems.

### 4.3. Limitations and Challenges

The proposed AWGWO algorithm, while demonstrating strong performance across various test functions and parameter estimation of the IM models, has certain limitations and challenges. One primary challenge is the increased computational complexity due to the adaptive weight mechanism. The dynamic adjustment of weights adds computational overhead, which may affect the algorithm’s efficiency for real-time applications. The adaptive mechanism could lead to longer convergence times, particularly for high-dimensional optimization problems. Another limitation is handling highly nonlinear, highly constrained, or dynamic optimization problems. Although the AWGWO algorithm shows excellent performance in offline parameter estimation and static optimization problems, its ability to adapt in real time could be limited. The proposed AWGWO may struggle to efficiently handle constraints that change over time or that are highly nonlinear in nature. Additionally, the algorithm may face challenges in finding the global optimum for multi-modal problems, especially when the exploration phase does not sufficiently spread the search space. Further developments, such as hybridization with other metaheuristics or incorporating other exploration mechanisms, including machine learning concepts, could be explored to address the challenges and increase the applicability of the AWGWO in real-world problems.

## 5. Conclusions

The parameter estimation of induction motor models using an adaptive version of the GWO algorithm is implemented and analyzed in this paper. The equivalent circuit parameter estimation of the IMs is converted into a multidimensional problem in which the equivalent circuit parameters of the induction motor are taken as problem dimensions or decision variables in the proposed method. Because of the complicated nature of the optimization problem, this strategy results in the formation of multimodal error landscapes for which it is extremely difficult to minimize associated cost functions. The proposed AWGWO algorithm, which is a relatively new variant of the original GWO algorithm, is used to estimate the parameters of the IMs. The AWGWO exhibits superior performance in multimodal situations, avoiding major defects such as premature convergence to sub-optimal solutions and other undesirable outcomes. The performance of the proposed AWGWO algorithm is compared with the different state-of-the-art algorithms and variants of GWO. It was found from the results that the proposed AWGWO algorithm is the best choice for the parameter estimation of IM problems. The AWGWO and other selected algorithms were applied and tested in two different test cases, such as the approximate circuit model and the exact circuit model. For both circuit models, the AWGWO algorithm produced better results than the other selected algorithms. In addition, the proposed algorithm is also validated for eight commercial induction motors to check the ability of the algorithm as a potential commercial tool. The convincing results of handling these complicated cases demonstrate that AWGWO outperforms the other algorithms, including different variants of the GWO. Therefore, AWGWO is an interesting alternative to conventional optimizers and may be commonly used for complex real-world problems.

In the future, the potential of the AWGWO algorithm could be further explored by extending its application to other types of electric machines and drives. Investigating its performance on larger datasets and under varying load conditions could provide deeper insights into its scalability and robustness. Moreover, integrating hybrid optimization techniques with AWGWO may improve convergence rates and precision. Finally, incorporating a machine learning-driven adaptive mechanism could enhance its adaptability to dynamic industrial environments. Further, the proposed AWGWO can also be extended for real-time parameter estimation and validated through hardware-in-the-loop simulations or experimental setups to assess its practical deployment in industrial applications. Hybrid approaches integrating AWGWO with deep learning models can also be explored for real-time parameter estimation and adaptive control in industrial applications.

## Figures and Tables

**Table 1:** Details of the traditional CEC benchmark functions.

Function	Dim	Range	*f*_min_
${TF}_{1}\left(x\right)=\sum_{i=1}^{n}\;x_{i}^{2}$	30	[−100, 100]	0
${TF}_{2}\left(x\right)=\sum_{i=1}^{n}\;\left|x_{i}\right|+\prod_{i=1}^{n}\;\left|x_{i}\right|$		[−10, 10]	
${TF}_{3}\left(x\right)=\sum_{i=1}^{n}\;{\left(\sum_{j-1}^{i}\;x_{j}\right)}^{2}$		[−100, 100]	
${TF}_{4}\left(x\right)={max}_{i}\left\{\left|x_{i}\right|,1⩽i⩽n\right\}$			
${TF}_{5}(x)=\sum_{i=1}^{n}\;[x_{i}^{2}-10cos(2\pi x_{i})+10]$		[−5.12, 5.12]	
${TF}_{6}(x)=-20exp\left(-0.2\sqrt{\frac{1}{n}\sum_{i=1}^{n}\;x_{i}^{2}}\right)-exp\left(\frac{1}{n}\sum_{i=1}^{n}\;cos(2\pi x_{i})\right)+20+e$		[−32, 32]	
${TF}_{7}(x)=\frac{1}{4000}\sum_{i=1}^{n}\;x_{i}^{2}-\prod_{i=1}^{n}\;cos\left(\frac{x_{i}}{\sqrt{i}}\right)+1$		[−600, 600]	
${TF}_{8}(x)=4x_{1}^{2}-2.1x_{1}^{4}+\frac{1}{3}x_{1}^{6}+x_{1}x_{2}-4x_{2}^{2}+4x_{2}^{4}$	2	[−5, 5]	−1.0316
${TF}_{9}(x)={\left(x_{2}-\frac{5.1}{4\pi^{2}}x_{1}^{2}+\frac{5}{\pi }x_{1}-6\right)}^{2}+10\left(1-\frac{1}{8\pi }\right)cosx_{1}+10$			0.398
${TF}_{10}(x)=-\sum_{i=1}^{10}\;{\left[(X-a_{i}){\left(X-a_{i}\right)}^{T}+c_{i}\right]}^{-1}$	4	[0, 10]	−10.5363

**Table 2:** Obtained results for the benchmark functions.

Functions	Metrics	AWGWO	GWO	LSGWO	DE	GSA	IGWO-DS	GWO-CM	PSO
${TF}_{1}$	Min	**4.63E-126**	2.48E-29	8.43E-04	1.70E-04	2.56E-01	3.85E-111	4.20E-87	4.37E+02
	Max	6.78E-115	1.81E-27	1.51E+00	6.41E-04	6.86E+01	1.32E-97	8.30E-75	1.42E+04
	Mean	6.78E-116	7.67E-28	5.46E-01	3.01E-04	1.01E+01	1.80E-98	1.55E-75	4.38E+03
	STD	2.14E-115	5.37E-28	5.88E-01	1.62E-04	2.07E+01	4.16E-98	3.03E-75	5.67E+03
	RT	0.112	0.088	0.153	0.273	0.113	0.181	2.628	0.161
	FRT	**1**	4	6	5	7	2	3	8
${TF}_{2}$	Min	**2.70E-65**	2.37E-17	1.12E-01	2.26E-03	7.54E-04	6.03E-57	4.66E-57	5.57E+00
	Max	3.55E-59	1.86E-16	3.24E+00	7.05E-03	5.82E-02	5.03E-51	3.15E-50	5.33E+01
	Mean	4.20E-60	1.00E-16	1.05E+00	4.44E-03	1.17E-02	6.01E-52	3.23E-51	2.61E+01
	STD	1.12E-59	6.33E-17	1.04E+00	1.20E-03	1.70E-02	1.58E-51	9.93E-51	1.34E+01
	RT	0.103	0.08	0.16	0.19	0.12	0.16	2.61	0.15
	FRT	**1**	4	7	5.4	5.6	2.5	2.5	8
${TF}_{3}$	Min	**1.69E-104**	1.26E-08	8.64E-01	3.18E+04	1.44E+03	9.36E-96	1.66E+04	9.96E+03
	Max	6.71E-93	2.79E-04	3.91E+01	4.61E+04	1.87E+04	6.06E-78	6.65E+04	5.19E+04
	Mean	6.72E-94	3.04E-05	2.30E+01	3.91E+04	7.45E+03	7.81E-79	4.96E+04	2.91E+04
	STD	2.12E-93	8.76E-05	1.10E+01	4.64E+03	5.01E+03	1.93E-78	1.68E+04	1.43E+04
	RT	0.22	0.18	0.24	0.27	0.29	0.29	2.66	0.25
	FRT	**1**	3	4	7	5.1	2	7.6	6.3
${TF}_{4}$	Min	2.93E-57	1.07E-07	7.17E-02	5.28E+00	2.34E+01	**1.98E-58**	6.18E-01	5.84E+01
	Max	4.86E-52	3.05E-06	1.29E+00	1.10E+01	5.32E+01	2.23E-48	8.48E+01	8.27E+01
	Mean	6.27E-53	1.05E-06	5.40E-01	7.66E+00	3.96E+01	3.00E-49	4.29E+01	7.08E+01
	STD	1.55E-52	1.11E-06	3.27E-01	1.80E+00	9.86E+00	6.89E-49	3.49E+01	6.46E+00
	RT	0.15	0.09	0.17	0.18	0.21	0.27	2.53	0.26
	FRT	**1.2**	3	4	5.3	6.4	1.8	6.6	7.7
${TF}_{5}$	Min	**0.00E+00**	5.68E-14	6.98E-03	1.40E+02	1.11E+01	**0.00E+00**	**0.00E+00**	1.07E+02
	Max	0.00E+00	1.10E+01	4.89E+01	1.67E+02	5.87E+01	0.00E+00	0.00E+00	1.74E+02
	Mean	0.00E+00	3.95E+00	1.85E+01	1.57E+02	3.58E+01	0.00E+00	0.00E+00	1.41E+02
	STD	0.00E+00	4.20E+00	1.92E+01	8.79E+00	1.87E+01	0.00E+00	0.00E+00	2.02E+01
	RT	0.12	0.09	0.15	0.18	0.17	0.19	2.58	0.17
	FRT	**1.65**	4	6.1	5	7.5	1.65	2.7	7.4
${TF}_{6}$	Min	**4.44E-16**	9.28E-14	1.20E-02	3.65E-03	7.39E-02	**4.44E-16**	**4.44E-16**	1.77E+01
	Max	4.44E-16	1.39E-13	3.50E+00	7.98E-03	2.03E+01	4.44E-16	7.55E-15	2.00E+01
	Mean	4.44E-16	1.09E-13	1.04E+00	5.00E-03	1.39E+01	4.44E-16	3.64E-15	1.97E+01
	STD	0.00E+00	1.28E-14	1.18E+00	1.35E-03	9.02E+00	0.00E+00	2.62E-15	7.02E-01
	RT	0.15	0.10	0.16	0.19	0.21	0.29	2.49	0.28
	FRT	**2.4**	3	5.7	5.1	7	**2.4**	**2.4**	8
${TF}_{7}$	Min	**0.00E+00**	**0.00E+00**	5.27E-06	2.88E-04	5.67E-01	**0.00E+00**	**0.00E+00**	6.31E+00
	Max	0.00E+00	1.76E-02	4.39E-02	4.64E-02	1.21E+00	0.00E+00	0.00E+00	2.31E+01
	Mean	0.00E+00	2.90E-03	1.61E-02	7.02E-03	9.23E-01	0.00E+00	0.00E+00	1.17E+01
	STD	0.00E+00	6.28E-03	1.52E-02	1.45E-02	2.02E-01	0.00E+00	0.00E+00	5.03E+00
	RT	0.14	0.11	0.17	0.15	0.19	0.21	2.58	0.18
	FRT	**1.2**	5.8	4.2	3.1	7.2	1.8	4.9	7.8
${TF}_{8}$	Min	**−1.0316**	**−1.0316**	**−1.0316**	**−1.0316**	**−1.0316**	**−1.0316**	**−1.0316**	**−1.0316**
	Max	−1.0316	−1.0316	−1.0316	−1.0316	−1.0315	−1.0316	−1.0316	−1.0316
	Mean	−1.0316	−1.0316	−1.0316	−1.0316	−1.0316	−1.0316	−1.0316	−1.0316
	STD	1.05E-16	1.05E-08	2.22E-16	0.00E+00	3.21E-05	8.18E-10	8.11E-10	7.40E-17
	RT	0.09	0.02	0.11	0.14	0.13	0.17	2.51	0.12
	FRT	**2.5**	6.2	**2.5**	**2.5**	8	5.9	5.9	**2.5**
${TF}_{9}$	Min	**0.3979**	**0.3979**	**0.3979**	**0.3979**	0.3980	**0.3979**	**0.3979**	**0.3979**
	Max	0.3979	0.3979	0.3979	0.3979	0.4056	0.3979	0.3979	0.3979
	Mean	0.3979	0.3979	0.3979	0.3979	0.4004	0.3979	0.3979	0.3979
	STD	0.00E+00	5.23E-06	0.00E+00	0.00E+00	2.46E-03	1.31E-05	7.24E-06	0.00E+00
	RT	0.07	0.01	0.09	0.11	0.11	0.15	2.60	0.11
	FRT	2.3	6.7	3.85	**1.4**	7.5	4.9	6.5	2.85
${TF}_{10}$	Min	**−10.5364**	−10.5360	**−10.5364**	**−10.5364**	−7.6146	−5.1280	−10.5362	**−10.5364**
	Max	−5.1285	−10.5333	−5.1285	−10.5364	−0.9424	−5.0997	−5.1223	−1.8595
	Mean	−8.3732	−10.5349	−8.9235	−10.5364	−4.7679	−5.1222	−8.6365	−7.4206
	STD	2.79E+00	8.02E-04	2.60E+00	1.87E-15	2.03E+00	8.67E-03	2.55E+00	4.05E+00
	RT	0.13	0.05	0.14	0.19	0.16	0.21	2.51	0.15
	FRT	3.45	4	3.8	**1.2**	6.9	6.9	5.2	4.55
Mean FRT		**1.77**	4.37	4.715	4.1	6.82	3.185	4.73	6.31

**Table 3:** Nameplate details of the motor under experimentation.

S. No.	Parameters	Specification
1	Current (*I_fl_*) in A	45
2	Voltage (*V_fl_*) in V	400
3	Power (*P_fl_*) in HP	40
4	Number of poles	4
5	Frequency (F) in Hz	50
6	Starting current (*I_st_*) in A	180
7	Full-load torque (*T_fl_*) in N-m	190
8	Maximum-load torque (*T_m_*) in N-m	370
9	Starting torque (*T_st_*) in N-m	260
10	Full-load slip (s*_fl_*) in %	9

**Table 4:** Parameters obtained by all selected algorithms for the approximate circuit model.

Algorithms	$R_{1}$ (Ω)	$R_{2}$ (Ω)	$X_{1}$ (Ω)	s	Fitness Function Value
PSO	0.1025	0.9874	0.5046	0.1048	1.0495 × 10^−7^
GSA	0.1156	0.9815	0.4988	0.1040	9.2153 × 10^−8^
GWO	0.1502	0.9759	0.4794	0.1011	8.8727 × 10^−8^
DE	0.5994	0.4943	0.2439	0.0608	4.8692 × 10^−8^
ABC [31]	1.693	0.759	1.526	NA	NA
BSFABC [31]	1.968	0.7984	1	NA	NA
EABC [31]	1.403	0.823	2.033	NA	NA
DBHABC [31]	1.382	0.751	1	NA	NA
AWGWO	**0.3919**	**0.9032**	**03480**	**0.0799**	**4.3342 × 10^−8^**

NA—not available.

**Table 5:** Estimated parameters and errors obtained by all selected algorithms of the approximate circuit model.

Algorithms	$T_{fl}$ (Nm)	$\mathrm{Error}\;\mathrm{in}\;T_{fl}$	$T_{m}$ (Nm)	$\mathrm{Error}\;\mathrm{in}\;T_{m}$	$T_{st}$ (Nm)	$\mathrm{Error}\;\mathrm{in}\;T_{st}$
PSO	189.8076	0.1924	370.1391	−0.1391	260.0900	−0.09
GSA	190.0097	−0.0097	369.7911	0.2089	259.9513	0.0487
GWO	189.9144	0.0856	369.8451	0.1549	259.9170	0.0830
DE	189.9132	0.0868	369.9557	0.0443	259.9310	0.0691
ABC [31]	194.774	−2.513	360.576	2.547	260.656	−0.252
BSFABC [31]	181.6591	4.39	343.0155	7.293	264.3033	1.655
EABC [31]	189.927	0.039	369.85	0.041	259.483	0.199
DBHABC [31]	189.953	0.0247	369.857	0.0386	260.556	0.2138
AWGWO	190.2889	−0.2889	370.3286	−0.3286	260.3685	−0.3685

**Table 6:** Statistical data of all selected algorithms for the approximate circuit model.

Algorithms	Min	Max	Average	STD	RT
PSO	1.049E-07	7.093E-06	2.573E-07	2.799E-06	**12.1744**
GSA	9.2153E-08	2.533E-07	1.284E-07	7.157E-08	14.7143
GWO	8.8727E-08	4.725E-02	9.450E-03	2.113E-02	15.6507
DE	4.8692E-08	**8.283E-08**	6.735E-08	7.378E-08	15.5205
AWGWO	**4.3342E-08**	1.884E-07	**5.485E-08**	**6.202E-08**	16.7774

**Table 7:** Computed parameters of the approximate circuit model by different variants of GWO.

Algorithms	$R_{1}$ (Ω)	$R_{2}$ (Ω)	$X_{1}$ (Ω)	s	Fitness Function Value
LSGWO	0.5964	0.5043	0.2463	0.0613	8.7309 × 10^−7^
GWO-CM	0.6000	0.4927	0.2437	0.0608	5.6671 × 10^−8^
IGWO-DS	0.3888	0.9149	0.3541	0.0678	4.7220 × 10^−8^
AWGWO	**0.3919**	**0.9032**	**0.3480**	**0.0799**	**4.3342 × 10^−8^**

**Table 8:** Estimated parameters and errors of the approximate circuit model by different variants of GWO.

Algorithms	$T_{fl}$ (Nm)	$\mathrm{Error}\;\mathrm{in}\;T_{fl}$	$T_{m}$ (Nm)	$\mathrm{Error}\;\mathrm{in}\;T_{m}$	$T_{st}$ (Nm)	$\mathrm{Error}\;\mathrm{in}\;T_{st}$
LSGWO	189.1575	0.8425	370.3082	−0.3082	261.4844	−1.4844
GWO-CM	189.8839	0.1161	369.8744	0.1256	259.8014	0.1986
IGWO-DS	189.8787	0.1213	370.1823	−0.1823	260.2970	−0.2970
AWGWO	190.2889	−0.2889	370.3286	−0.3286	260.2685	−0.2685

**Table 9:** Statistical analysis of the approximate circuit model by different variants of GWO.

Algorithms	Min	Max	Average	STD	RT
LSGWO	8.7309E-07	4.725E-02	9.451E-03	2.113E-02	17.0214
GWO-CM	5.6671E-08	3.178E-07	1.609E-07	1.212E-07	23.7065
IGWO-DS	4.7220E-08	4.339E-07	1.472E-07	1.724E-07	35.2150
AWGWO	**4.3342E-08**	**1.884E-07**	**5.485E-08**	**6.202E-08**	**16.7774**

**Table 10:** Parameters obtained by all selected algorithms for the exact circuit model.

Algorithms	$R_{1}$ (Ω)	$X_{1}$ (Ω)	$R_{2}$ (Ω)	$X_{2}$ (Ω)	$X_{m}$ (Ω)	s (%)	Fitness
PSO	0.1819	0.1279	0.4413	0.9994	5.8727	0.0983	4.1630 × 10^−7^
GSA	0.4263	0.2841	0.3050	0.4894	7.3919	0.0768	2.5620 × 10^−7^
GWO	0.4387	0.2735	0.2873	0.4437	4.7941	0.0756	2.4504 × 10^−7^
DE	0.4372	0.2894	0.2987	0.4632	7.1306	0.0758	1.3056 × 10^−7^
GA [22]	0.4875	0.3264	0.3556	0.3556	6.6072	NA	3.5000 × 10^−3^
SFLA [22]	0.3437	0.3360	0.4345	0.4345	6.2629	NA	1.1000 × 10^−3^
MSFLA [22]	0.2707	0.3573	0.4773	0.4773	7.5432	NA	2.8000 × 10^−4^
ABC [31]	1.732	0.1	0.803	1.44	400	NA	NA
BSFABC [31]	1.508	1.84	0.892	0.12	400	NA	NA
EABC [31]	1.403	0.1	0.824	1.93	400	NA	NA
DBHABC [31]	1.217	0.10	0.773	0.10	400	NA	NA
AWGWO	**0.3977**	**0.2357**	**0.2981**	**0.5150**	**3.4079**	**0.0794**	**1.1026 × 10^−8^**

NA—not available.

**Table 11:** Estimated parameters and errors of the exact circuit model obtained by all algorithms.

Algorithms	$T_{fl}$ (Nm)	$\mathrm{Error}\;\mathrm{in}\;T_{fl}$	$T_{m}$ (Nm)	$\mathrm{Error}\;\mathrm{in}\;T_{m}$	$T_{st}$ (Nm)	$\mathrm{Error}\;\mathrm{in}\;T_{st}$	pf	Error in pf
PSO	189.8591	0.1409	370.0899	−0.0899	260.2098	−0.2098	0.8004	−4.1427 × 10^−4^
GSA	189.7253	0.2747	370.0843	−0.0843	260.1523	−0.1523	0.7993	6.6217 × 10^−4^
GWO	190.1486	−0.1486	370.4367	−0.4367	260.1793	−0.1793	0.8001	−1.1833 × 10^−4^
DE	189.9056	0.0944	370.0152	−0.0152	260.1370	−0.1370	0.8000	−4.2484 × 10^−5^
GA [22]	192.7880	2.7880	354.0920	15.9080	268.0160	8.0160	0.8170	0.0170
SFLA [22]	195.1060	5.1060	368.0360	1.9640	262.4670	2.4670	0.7860	0.0140
MSFLA [22]	192.1970	2.1970	373.852	3.8520	261.6870	1.6870	0.7995	0.0005
ABC [31]	185.846	2.186	353.752	4.391	261.725	−0.664	NA	NA
BSFABC [31]	174.979	7.906	358.528	3.101	266.194	−2.382	NA	NA
EABC [31]	189.752	0.131	370.075	−0.02	260.013	−0.005	NA	NA
DBHABC [31]	190.734	0.374	369.081	0.248	259.991	0.0034	NA	NA
AWGWO	189.7910	0.2090	370.1063	−0.1063	260.2997	−0.2997	0.8003	−3.1990 × 10^−4^

NA—not available.

**Table 12:** Statistical analysis of all selected algorithms for the exact circuit model.

Algorithms	Min	Max	Average	STD	RT
PSO	4.1630E-07	2.812E-06	9.313E-07	1.055E-06	**14.1569**
GSA	2.5620E-07	9.683E-07	3.011E-07	1.241E-07	18.5698
GWO	2.4504E-07	6.607E-07	2.120E-07	1.162E-07	19.9856
DE	1.3056E-07	**3.004E-07**	**1.202E-07**	4.558E-08	16.6944
AWGWO	**1.1026E-08**	4.582E-07	9.453E-07	**1.906E-09**	20.2568

**Table 13:** Parameters obtained by different variants of GWO for the exact circuit model.

Algorithms	$R_{1}$ (Ω)	$X_{1}$ (Ω)	$R_{2}$ (Ω)	$X_{2}$ (Ω)	$X_{m}$ (Ω)	s	Fitness Function Value
LSGWO	0.3191	0.2238	0.3699	0.7352	9.9167	0.0866	8.8038 × 10^−7^
GWO-CM	0.2756	0.1129	0.3066	0.6423	0.9465	0.0902	2.4099 × 10^−7^
IGWO-DS	0.1902	0.1369	0.4437	0.9990	9.5711	0.0976	8.0533 × 10^−8^
AWGWO	**0.3977**	**0.2357**	**0.2981**	**0.5150**	**3.4079**	**0.0794**	**1.1026 × 10^−8^**

**Table 14:** Estimated parameters and errors of the exact circuit model obtained by different variants of GWO.

Algorithms	$T_{fl}$ (Nm)	$\mathrm{Error}\;\mathrm{in}\;T_{fl}$	$T_{m}$ (Nm)	$\mathrm{Error}\;\mathrm{in}\;T_{m}$	$T_{st}$ (Nm)	$\mathrm{Error}\;\mathrm{in}\;T_{st}$	pf	Error in pf
LSGWO	189.5157	0.4843	369.8549	0.1451	259.6347	0.3653	0.8022	−0.00220
GWO-CM	189.3420	0.6580	369.8387	0.1613	260.5639	−0.5639	0.7997	2.5406 × 10^−6^
IGWO-DS	190.2779	−0.2779	370.1230	−0.1230	259.9000	0.1000	0.8002	−1.6099 × 10^−4^
AWGWO	189.7910	0.2090	370.1063	−0.1063	260.2997	−0.2997	0.8003	−3.1990 × 10^−4^

**Table 15:** Statistical data obtained by all variants of GWO for the exact circuit model.

Algorithm	Min	Max	Average	STD	RT
LSGWO	8.089E-07	5.553E-06	3.040E-06	1.871E-06	20.4125
GWO-CM	1.579E-07	8.094E-07	4.855E-07	2.766E-07	24.1456
IGWO-DS	8.053E-08	**6.094E-07**	2.713E-07	2.105E-07	26.7845
AWGWO	**7.505E-08**	8.158E-07	**1.037E-07**	**3.092E-08**	**20.2568**

**Table 16:** Specifications of commercial induction motors.

Specifications	Motor 1	Motor 2	Motor 3	Motor 4	Motor 5	Motor 6	Motor 7	Motor 8
*V_fl_* in V	400	415	415	415	230	230	230	230
*P_fl_* in kW	37	30	22	15	11	7.5	3	3
*F* in Hz	50	50	50	50	50	50	50	50
*η_fl_*	0.962	0.925	0.912	0.85	0.912	0.901	0.871	0.871
*I*_fl_ in A	64	52.8	42	31.2	19.2	13.1	9.3	10.2
cosφ	0.87	0.87	0.83	0.81	0.91	0.9	0.9	0.85
Speed in RPM	1470	1470	970	730	2943	2909	2896	2915
*T*_fl_ in Nm	240.46	195	218	197	36	24.6	9.9	9.83
*I*_st_/*I*_fl_	6	6.8	6.4	6	8	8.3	8.4	8.3
*T*_m_/*T*_fl_	2.5	2.7	2.8	2.6	3.6	3.9	3.9	3.3
*T*_st_/*T*_fl_	2.2	2.4	2.3	2.1	2.6	3	3.2	2

**Table 17:** Parameters obtained by all selected algorithms for the commercial motors.

Motor No.	Algorithms	$R_{1}$ (Ω)	$X_{1}$ (Ω)	$R_{2}$ (Ω)	$X_{2}$ (Ω)	$X_{m}$ (Ω)	s	Best Fitness
Motor 1	AWGWO	**0.2656**	**0.1275**	**0.2913**	**0.3417**	**3.7763**	**0.0866**	**8.85E-24**
	GWO	0.2490	0.1213	0.3036	0.3771	3.8691	0.0891	7.58E-08
	PSO	0.2683	0.1272	0.2982	0.3411	3.7393	0.0887	4.60E-05
	GSA	0.2539	0.1290	0.3049	0.3725	5.3651	0.0883	1.70E-06
	DE	0.2903	0.1547	0.2872	0.3020	11.0000	0.0828	4.21E-17
Motor 2	AWGWO	**0.3372**	**0.1644**	**0.3623**	**0.3999**	**5.2755**	**0.0798**	**3.310E-12**
	GWO	0.3061	0.1528	0.3863	0.4680	5.6533	0.0834	2.513E-07
	PSO	0.2215	0.1000	0.4199	0.5914	1.9850	0.0929	7.086E-05
	GSA	0.3202	0.1274	0.3497	0.4086	2.2396	0.0816	2.530E-06
	DE	0.3137	0.1651	0.3900	0.4626	9.9066	0.0826	3.737E-12
Motor 3	AWGWO	**0.2471**	**0.1533**	**0.5137**	**0.8643**	**6.6287**	**0.0783**	**1.580E-22**
	GWO	0.2189	0.1359	0.5313	0.9157	5.8951	0.0804	7.973E-08
	PSO	0.4901	0.1447	0.3087	0.3260	1.7213	0.0598	9.839E-04
	GSA	0.4390	0.2538	0.3795	0.4583	6.2687	0.0630	2.320E-06
	DE	0.4811	0.2814	0.3594	0.3668	7.4043	0.0601	1.502E-10
Motor 4	AWGWO	**0.5997**	**0.3344**	**0.6000**	**0.8473**	**4.7252**	**0.0720**	**0.000E+00**
	GWO	0.5974	0.3179	0.5886	0.8336	3.9921	0.0721	9.837E-08
	PSO	0.5661	0.3631	0.6000	0.9318	9.3455	0.0665	6.946E-04
	GSA	0.5973	0.3231	0.5901	0.8372	4.2305	0.0718	3.497E-06
	DE	0.5995	0.3341	0.6000	0.8475	4.7147	0.0720	4.386E-14
Motor 5	AWGWO	**0.1921**	**0.1000**	**0.2000**	**0.3377**	**10.0000**	**0.0482**	**3.857E-03**
	GWO	0.2142	0.1000	1.0000	0.3000	4.7803	0.2489	8.289E-03
	PSO	0.1981	0.1000	1.0000	0.3000	2.1635	0.2622	1.033E-02
	GSA	0.1916	0.1000	0.2000	0.3376	8.5397	0.0484	4.076E-03
	DE	0.2145	0.1000	1.0000	0.3000	4.8941	0.2487	8.289E-03
Motor 6	AWGWO	**0.2194**	**0.1004**	**0.2710**	**0.5039**	**9.9975**	**0.0436**	**1.671E-16**
	GWO	0.3122	0.1000	1.0000	0.3000	1.4261	0.1937	8.718E-03
	PSO	0.2927	0.1000	0.2000	0.3000	1.6483	0.0374	5.155E-04
	GSA	0.3353	0.1044	1.0000	0.3000	2.1043	0.1841	9.982E-03
	DE	0.3165	0.1410	0.2140	0.3010	9.7443	0.0358	3.456E-12
Motor 7	AWGWO	**0.8018**	**0.2887**	**0.5615**	**0.6710**	**7.2870**	**0.0402**	**6.353E-29**
	GWO	0.8173	0.1986	0.5185	0.5971	3.5970	0.0396	9.665E-08
	PSO	0.7424	0.1916	0.6078	0.8508	5.0526	0.0433	7.374E-04
	GSA	0.6341	0.2134	0.6541	0.9984	4.8126	0.0465	7.619E-06
	DE	0.6724	0.2725	0.6548	0.9660	9.9047	0.0447	5.194E-12
Motor 8	AWGWO	**0.6000**	**0.1000**	**0.3870**	**1.0000**	**1.1774**	**0.0354**	**1.394E-03**
	GWO	0.6000	0.1000	0.3872	1.0000	1.1760	0.0355	1.395E-03
	PSO	0.6000	0.1894	0.3797	1.0000	1.6755	0.0330	3.442E-03
	GSA	0.6000	0.1194	0.3880	0.9998	1.2558	0.0353	1.660E-03
	DE	**0.6000**	**0.1000**	**0.3870**	**1.0000**	**1.1774**	**0.0354**	**1.394E-03**

**Table 18:** Estimated parameters and errors of the exact circuit model obtained by all algorithms for the commercial motors.

Motor No.	Algorithms	$T_{fl}$ (Nm)	$\mathrm{Error}\;\mathrm{in}\;T_{fl}$	$T_{m}$ (Nm)	$\mathrm{Error}\;\mathrm{in}\;T_{m}$	$T_{st}$ (Nm)	$\mathrm{Error}\;\mathrm{in}\;T_{st}$	pf	Error in pf
Motor 1	AWGWO	240.4615	1.48E-03	601.1370	1.30E-02	529.0188	6.80E-03	0.8700	1.34E-09
	GWO	240.2838	1.76E-01	600.7601	3.90E-01	528.2561	7.56E-01	0.8697	2.93E-04
	PSO	240.2162	2.44E-01	598.4854	2.66E+00	531.4233	2.41E+00	0.8719	1.86E-03
	GSA	244.4188	3.96E+00	598.7888	2.36E+00	526.4271	2.58E+00	0.8723	2.30E-03
	DE	240.4639	3.88E-03	601.1588	8.76E-03	529.0209	8.94E-03	0.8700	5.21E-06
Motor 2	AWGWO	195.0000	4.42E-05	526.4983	1.65E-03	468.0011	1.14E-03	0.8700	6.61E-07
	GWO	194.9206	7.94E-02	526.3572	1.43E-01	467.5901	4.10E-01	0.8701	1.20E-04
	PSO	195.0978	9.78E-02	526.2919	2.08E-01	467.1433	8.57E-01	0.8629	7.13E-03
	GSA	197.5206	2.52E+00	528.4356	1.94E+00	469.5004	1.50E+00	0.8725	2.52E-03
	DE	194.9999	6.13E-05	526.8172	3.17E-01	468.4592	4.59E-01	0.8697	2.52E-04
Motor 3	AWGWO	218.0000	3.79E-09	610.3999	7.60E-05	501.4001	5.56E-05	0.8300	7.97E-09
	GWO	217.8997	1.00E-01	610.8046	4.05E-01	502.0696	6.70E-01	0.8295	4.65E-04
	PSO	217.0364	9.64E-01	609.3855	1.01E+00	502.5774	1.18E+00	0.8557	2.57E-02
	GSA	217.0041	9.96E-01	610.4461	4.61E-02	500.2396	1.16E+00	0.8303	3.21E-04
	DE	218.5128	5.13E-01	610.5979	1.98E-01	501.1453	2.55E-01	0.8309	8.96E-04
Motor 4	AWGWO	197.0000	0.00E+00	512.2000	0.00E+00	413.7000	0.00E+00	0.8100	0.00E+00
	GWO	196.9198	8.02E-02	512.1304	6.96E-02	413.6990	1.04E-03	0.8096	3.77E-04
	PSO	196.7801	2.20E-01	523.2646	1.11E+01	407.4978	6.20E+00	0.8089	1.13E-03
	GSA	195.4200	1.58E+00	513.2434	1.04E+00	413.9373	2.37E-01	0.8103	3.09E-04
	DE	196.9996	4.48E-04	512.1962	3.80E-03	413.6958	4.23E-03	0.8100	4.31E-07
Motor 5	AWGWO	36.0001	1.00E-04	123.6633	5.94E+00	95.7570	2.16E+00	0.8781	3.19E-02
	GWO	35.9873	1.27E-02	122.0890	7.51E+00	99.7156	6.12E+00	0.8865	2.35E-02
	PSO	36.1292	1.29E-01	121.4679	8.13E+00	97.8295	4.23E+00	0.8500	6.00E-02
	GSA	35.8029	1.97E-01	123.5587	6.04E+00	95.6348	2.03E+00	0.8749	3.51E-02
	DE	36.0000	5.32E-09	122.0063	7.59E+00	99.6937	6.09E+00	0.8874	2.26E-02
Motor 6	AWGWO	24.6000	2.72E-07	95.9400	5.65E-07	73.8000	2.31E-07	0.9000	8.38E-10
	GWO	24.6049	4.95E-03	90.6112	5.33E+00	78.8339	5.03E+00	0.8718	2.82E-02
	PSO	24.6489	4.89E-02	96.0609	1.21E-01	73.5365	2.64E-01	0.8799	2.01E-02
	GSA	24.6442	4.42E-02	90.0774	5.86E+00	79.6408	5.84E+00	0.8986	1.37E-03
	DE	24.5963	3.71E-03	95.9354	4.58E-03	73.8004	3.82E-04	0.9000	8.86E-06
Motor 7	AWGWO	9.9000	3.55E-15	38.6100	1.71E-13	31.6800	1.74E-13	0.9000	3.33E-15
	GWO	9.9086	8.63E-03	38.5871	2.29E-02	31.6408	3.92E-02	0.9003	2.69E-04
	PSO	9.8762	2.38E-02	38.4415	1.68E-01	31.8248	1.45E-01	0.9237	2.37E-02
	GSA	9.7641	1.36E-01	38.5272	8.28E-02	31.6977	1.77E-02	0.8997	2.71E-04
	DE	9.8990	9.58E-04	38.6100	1.06E-05	31.6956	1.56E-02	0.9000	3.32E-06
Motor 8	AWGWO	9.8300	3.21E-07	33.0709	6.32E-01	19.6600	4.04E-07	0.8229	7.08E-03
	GWO	9.8488	1.88E-02	33.0906	6.52E-01	19.6623	2.27E-03	0.8231	6.93E-03
	PSO	9.8031	2.69E-02	33.8048	1.37E+00	19.5684	9.16E-02	0.8156	1.44E-02
	GSA	9.8129	1.71E-02	33.1536	7.15E-01	19.6750	1.50E-02	0.8209	9.09E-03
	DE	9.8300	7.25E-09	33.0709	6.32E-01	19.6600	7.99E-09	0.8229	7.08E-03

**Table 19:** Statistical analysis of all selected algorithms for the commercial motors.

Motor No.	Algorithms	Min	Max	Mean	STD	RT
Motor 1	AWGWO	**8.846E-24**	**6.676E-10**	**1.672E-10**	**3.336E-10**	9.25
	GWO	7.582E-08	5.956E-07	3.186E-07	2.133E-07	**8.86**
	PSO	4.603E-05	8.836E-03	5.431E-03	3.880E-03	11.03
	GSA	1.704E-06	7.986E-03	2.023E-03	3.975E-03	9.62
	DE	4.205E-17	2.550E-08	6.485E-09	1.268E-08	10.10
Motor 2	AWGWO	**3.310E-12**	**2.680E-11**	**1.335E-11**	**1.054E-11**	8.82
	GWO	2.513E-07	3.798E-07	3.290E-07	5.817E-08	**8.11**
	PSO	7.086E-05	5.525E-02	1.667E-02	2.586E-02	9.41
	GSA	2.530E-06	9.289E-05	2.658E-05	4.427E-05	9.56
	DE	3.737E-12	4.657E-09	1.759E-09	2.041E-09	9.12
Motor 3	AWGWO	**1.580E-22**	**2.793E-14**	**6.988E-15**	**1.396E-14**	10.75
	GWO	7.973E-08	7.800E-07	3.757E-07	3.084E-07	**10.36**
	PSO	9.839E-04	2.017E-02	6.861E-03	8.995E-03	12.74
	GSA	2.320E-06	2.071E-04	5.787E-05	9.958E-05	11.17
	DE	1.502E-10	5.969E-09	3.135E-09	2.863E-09	11.37
Motor 4	AWGWO	**0.000E+00**	**3.969E-32**	**1.932E-32**	**1.622E-32**	6.41
	GWO	9.837E-08	5.483E-05	1.407E-05	2.717E-05	**5.96**
	PSO	6.946E-04	6.841E-03	2.627E-03	2.847E-03	7.12
	GSA	3.497E-06	2.237E-01	5.596E-02	1.118E-01	7.08
	DE	4.386E-14	2.237E-01	5.591E-02	1.118E-01	6.45
Motor 5	AWGWO	**3.857E-03**	**8.289E-03**	**4.965E-03**	2.216E-03	6.23
	GWO	8.289E-03	8.306E-03	8.297E-03	7.071E-06	**5.77**
	PSO	1.033E-02	3.703E-02	2.265E-02	1.172E-02	7.62
	GSA	4.076E-03	8.904E-03	7.489E-03	2.284E-03	6.90
	DE	8.289E-03	8.289E-03	8.289E-03	**5.925E-18**	7.18
Motor 6	AWGWO	**1.671E-16**	8.742E-03	**2.186E-03**	4.371E-03	6.02
	GWO	8.718E-03	**8.719E-03**	8.719E-03	**5.621E-07**	**5.56**
	PSO	5.155E-04	1.426E-02	6.988E-03	7.021E-03	7.80
	GSA	9.982E-03	1.397E-02	1.285E-02	1.915E-03	6.50
	DE	3.456E-12	8.902E-03	6.590E-03	4.394E-03	6.86
Motor 7	AWGWO	**6.353E-29**	**1.656E-15**	**4.305E-16**	**8.169E-16**	6.17
	GWO	9.665E-08	4.159E-07	2.432E-07	1.318E-07	**5.74**
	PSO	7.374E-04	7.423E-03	3.281E-03	3.047E-03	7.32
	GSA	7.619E-06	1.979E-05	1.202E-05	5.538E-06	6.81
	DE	5.194E-12	2.724E-07	7.235E-08	1.335E-07	6.21
Motor 8	AWGWO	**1.394E-03**	2.014E-01	5.139E-02	1.000E-01	9.66
	GWO	1.395E-03	2.014E-01	5.140E-02	1.000E-01	**8.82**
	PSO	3.442E-03	**3.355E-02**	**2.239E-02**	**1.322E-02**	20.62
	GSA	1.660E-03	2.073E-01	1.081E-01	1.114E-01	8.79
	DE	1.394E-03	2.014E-01	1.014E-01	1.155E-01	9.62

## Data Availability

The data related to this study are included in the article. Additional related data may be provided upon reasonable request to the corresponding author.
